# A genome-wide genetic screen uncovers determinants of human pigmentation

**DOI:** 10.1126/science.ade6289

**Published:** 2023-08-11

**Authors:** Vivek K. Bajpai, Tomek Swigut, Jaaved Mohammed, Sahin Naqvi, Martin Arreola, Josh Tycko, Tayne C. Kim, Jonathan K. Pritchard, Michael C. Bassik, Joanna Wysocka

**Affiliations:** 1 Department of Chemical and Systems Biology, Stanford University School of Medicine, Stanford, CA 94305, USA.; 2 Institute for Stem Cell Biology and Regenerative Medicine, Stanford University School of Medicine, Stanford, CA 94305, USA.; 3 School of Chemical, Biological, and Materials Engineering, The University of Oklahoma, Norman, OK, 73019, USA.; 4 Department of Genetics, Stanford University, Stanford, CA 94305, USA.; 5 Department of Biology, Stanford University, Stanford, CA 94305, USA.; 6 Chemistry, Engineering, and Medicine for Human Health (ChEM-H), Stanford University, Stanford, CA 94305, USA.; 7 Program in Cancer Biology, Stanford University School of Medicine, Stanford, CA 94305 USA.; 8 Department of Developmental Biology, Stanford University School of Medicine, Stanford, CA 94305, USA.; 9 Howard Hughes Medical Institute, Stanford University School of Medicine, Stanford, CA 94305, USA.

## Abstract

Skin color, one of the most diverse human traits, is determined by the quantity, type, and distribution of melanin. In this study, we leveraged the light-scattering properties of melanin to conduct a genome-wide screen for regulators of melanogenesis. We identified 169 functionally diverse genes that converge on melanosome biogenesis, endosomal transport, and gene regulation, of which 135 represented previously unknown associations with pigmentation. In agreement with their melanin-promoting function, the majority of screen hits were up- regulated in melanocytes from darkly pigmented individuals. We further unraveled functions of KLF6 as a transcription factor that regulates melanosome maturation and pigmentation in vivo, and of the endosomal trafficking protein COMMD3 in modulating melanosomal pH. Our study reveals a plethora of melanin-promoting genes, with broad implications for human variation, cell biology, and medicine.

## One Sentence Summary

Genetic screen uncovers genes involved in human melanogenesis, many of which are differentially expressed in individuals of diverse skin color.

Skin and hair color are genetically derived traits that are highly variable be- tween and within human populations and determined by the quantity, type, and distribution of melanin (1-3). Melanocytes developmentally originate from the embryonic neural crest cells, reside in the epidermis, and synthesize melanin within subcellular lysosome- related organelles called melanosomes (4, 5). Melanosomes undergo characteristic stages of maturation during melanin synthesis in which stage I melanosomes contain intraluminal vesicles, stage II melanosomes deposit PMEL fibrils on which melanogenesis takes place, and stage III and IV melanosomes represent partially and fully melanized melanosomes, respectively (fig. S1A) (4, 5). Maturing melanosomes are transported extracellularly to the surrounding epidermal keratinocytes, which results in skin and hair pigmentation (fig. S1A) (1-5).

Key insights into human pigmentation have come from the mapping of genes involved in hypo- and hyperpigmentation diseases, and from candidate gene and genome-wide association studies (GWASs) of normal-range skin and hair color variation in human populations(1-3). Furthermore, studies in model organisms—in particular, those of mouse coat color—have revealed genes and pathways involved in pigmentation, many of which converge on the melanin synthesis pathway (1-3, 6). Nonetheless, GWASs that have estimated the cumulative contribution of major skin color genetic variants identified in a given human population could only explain a relatively small (23 to 35%) fraction of skin color variation in the analyzed population (2, 7-10). Similarly, using a GWAS summary statistic-based method (11), we estimated that only 15.2% of skin color variation in white British individuals from the UK Biobank (UKBB) could be explained by the set of genomic regions corresponding to the genome-wide significant skin color GWAS hits from this population. Taken together, these observations suggest that although key loci that control pigmentation in our species have been found, other contributing loci remain to be discovered.

Melanin is a heterogenous and structurally ill-defined biopolymer (12) that encompasses two forms, namely black or brown eumelanin and red or yellow pheomelanin. The quantity and type of produced melanin determines its physicochemical properties such as high refractive index, which forms the basis of melanin’s characteristic light-absorbance and scattering properties (12). These are thought to be critical for protecting the skin from sun exposure-related damage. Light scattering measured as a side-scatter (SSC, 90° scatter) parameter in flow cytometry reflects the internal complexity of the cells (13). We investigated the extent to which changes in cellular melanin content influence light scattering measured as SSC and whether SSC can be used to capture dynamic changes in the melanin levels upon inducing genetic perturbations. In this study, we demonstrate that cellular melanin concentration indeed determines light-scattering properties of pigment cells. Taking advantage of this feature and using SSC as a proxy for melanin levels, we performed a CRISPR-Cas9– based genetic screen to identify regulators of human pigmentation, which led to the discovery of 169 candidate genes with promelanogenic function. We validated a subset of the screen hits, confirming their transcriptional up-regulation in primary melanocytes from the dark-skin individuals and their involvement in different stages of melanosome biogenesis. Furthermore, through follow-up functional studies, we uncovered previously uncharacterized roles for transcription factor KLF6 in melanosome maturation and pigmentation in vivo, and a function for the endosomal trafficking protein COMMD3 in modulating melanosomal pH.

## Melanin content of the pigment cells can be reliably monitored by measuring their side scattering properties

**Fig.1.**
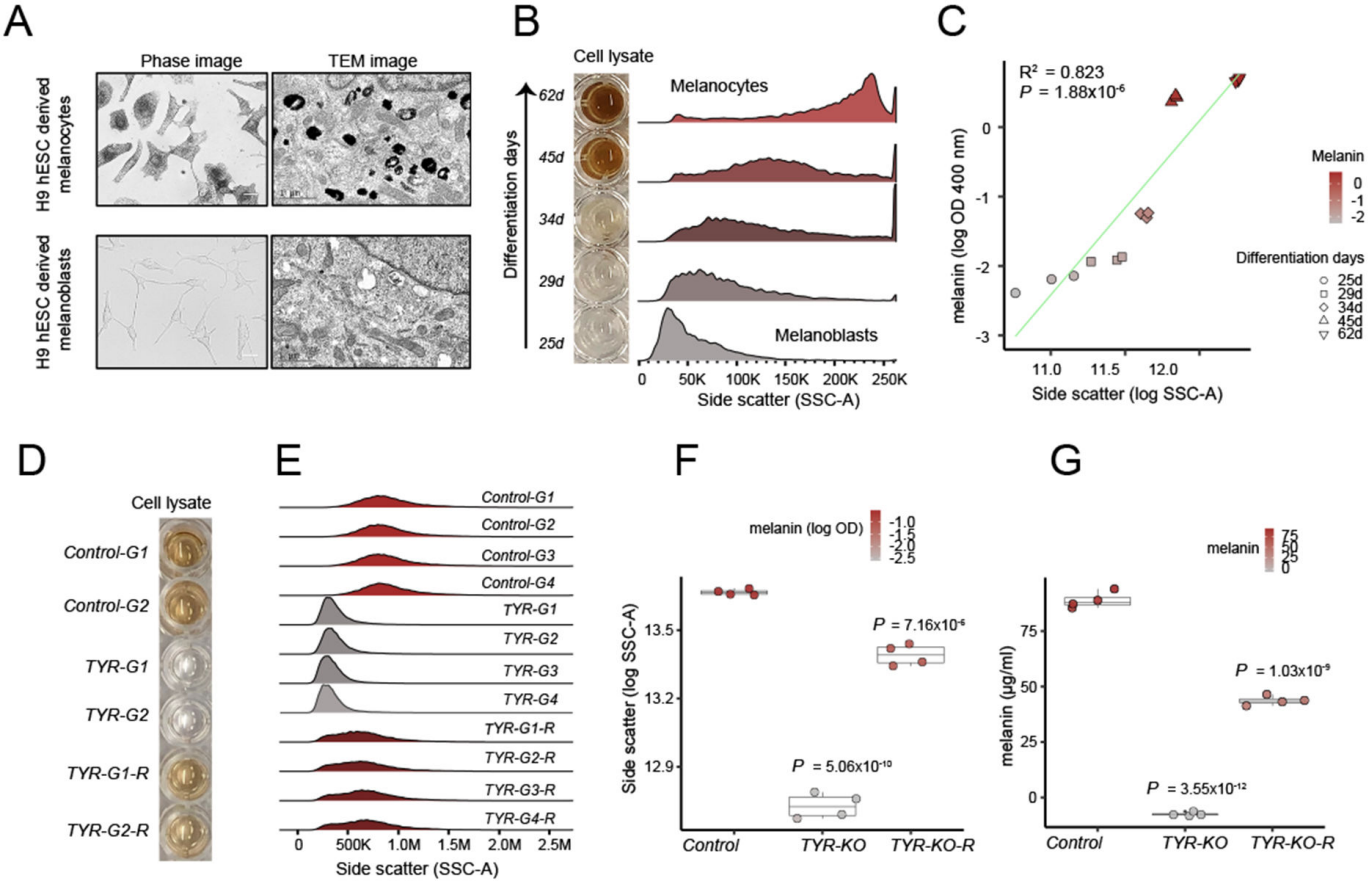
Melanin concentration determines light scattering property of pigment cells. (**A**) Differentiation of H9 human embryonic stem cell (hESC)– derived melanoblasts (bottom) into melanocytes (top) results in increased melanized stage III to IV melanosomes as shown by phase and transmission electron microscopy (n = 3 independent differentiations). Scale bars; phase images, 25 μm; TEM images, 1 μm. (**B**) Increased melanin concentration during melanocyte differentiation from hESC (days of differentiation are indicated on the left) is accompanied by an increase in light scattering as measured by the SSC parameter of flow cytometer. (**C**) Relationship between melanin (log OD 400 nm) and SSC as modeled by linear regression. R^2^ = 0.823, P = 1.88 × 10^−6^. (**D**) CRISPR-Cas9–mediated loss of TYR gene makes pigmented MNT-1 melanoma cells amelanotic and reexpression of TYR transgene recovers the pigmentation (n = 4 sgRNAs treatments). Cell lysates from two representative delete-and- rescue experiments are shown. (**E**) TYR loss significantly reduces the SSC, and TYR re-expression restores it. Four different sgRNAs against TYR and control sgRNAs are labeled as G1, G2, G3 and G4. (**F**) Boxplot showing SSC (log SSC) changes in response to TYR loss and rescue in comparison with control-edited cells. The color within the dots corresponds to total melanin (log OD at 400 nm). Box plots show median and IQR; whiskers are 1.5x interquartile range (IQR). Significance was tested for control, TYR-KO, and TYR-KO-R groups with analysis of variance (ANOVA), followed by a two-sided Welch t test with Benjamini and Hochberg (BH) correction. P values shown are relative to control. (**G**) Total melanin concentration calculated for loss and rescue of TYR (tyrosinase) experiment (n = 4 sgRNAs treatment). Box plots show median melanin concentration (micrograms per milliliter) and IQR; whiskers are 1.5x IQR. Significance was tested for four sgRNA and the TYR rescue treatment with ANOVA, followed by a two-sided Welch t-test with BH correction. P values shown are relative to control.

To establish a link between melanin content and SSC, we used two different cellular models of human melanogenesis. First, we investigated the relationship between SSC and pigmentation in a developmental context by modeling melanogenesis by using pluripotent stem cells (fig. S1B) (14). To this end, we differentiated SOX10::GFP-reporter H9 human embryonic stem cells to SOX10^+^ neural crest cells, then toward unpigmented melanocyte precursors called melanoblasts, and last into mature pigmented melanocytes (fig. S1, C and D). During the maturation of melanoblasts to melanocytes, the expression of melanogenic genes (e.g., OCA2, TYR, SLC45A2, OA1, and PMEL) was strongly up-regulated (fig. S1G), melanin content increased (fig. S1E, F and H), and pigmented melanosomes gradually accumulated (Fig. 1A and fig. S1H). We observed that an increase in melanin content was accompanied by an increase in SSC (Fig. 1B and C) and that there was a strong linear correlation between SSC and melanin content (coefficient of determination, R^2^ = 0.82; P = 1.88 × 10^−6^) (Fig. 1C).

In an orthogonal approach to establishing the relationship between SSC and pigmentation, we used the MNT-1 melanoma cell line, which is transcriptionally similar to normal primary human melanocytes (15), and has been previously used as a model to study melanogenesis (16-19). Using several independent RNA guides, we deleted the key melanogenic gene tyrosinase (TYR) in MNT-1 cells, which resulted in loss of pigmentation and decreased melanin absorbance measurements (Fig. 1D, G and fig. S1I). Loss of melanin was accompanied by a significant decrease in SSC (Fig. 1E, F). Transmission electron microscopy (TEM) images of TYR mutant cells showed a preponderance of stage II unpigmented melanosomes and a lack of stage III or IV melanized melanosomes, which thus provided evidence that the melanosome maturation state is directly linked to changes in SSC (fig. S1I). Furthermore, overexpression of wild-type TYR restored both pigmentation and SSC (Fig. 1D to G). Together, these experiments establish that perturbations in melanosome biogenesis of pigment cells can be monitored by measuring SSC.

## A genome-wide CRISPR screen to identify melanin promoting genes

**Fig. 2.**
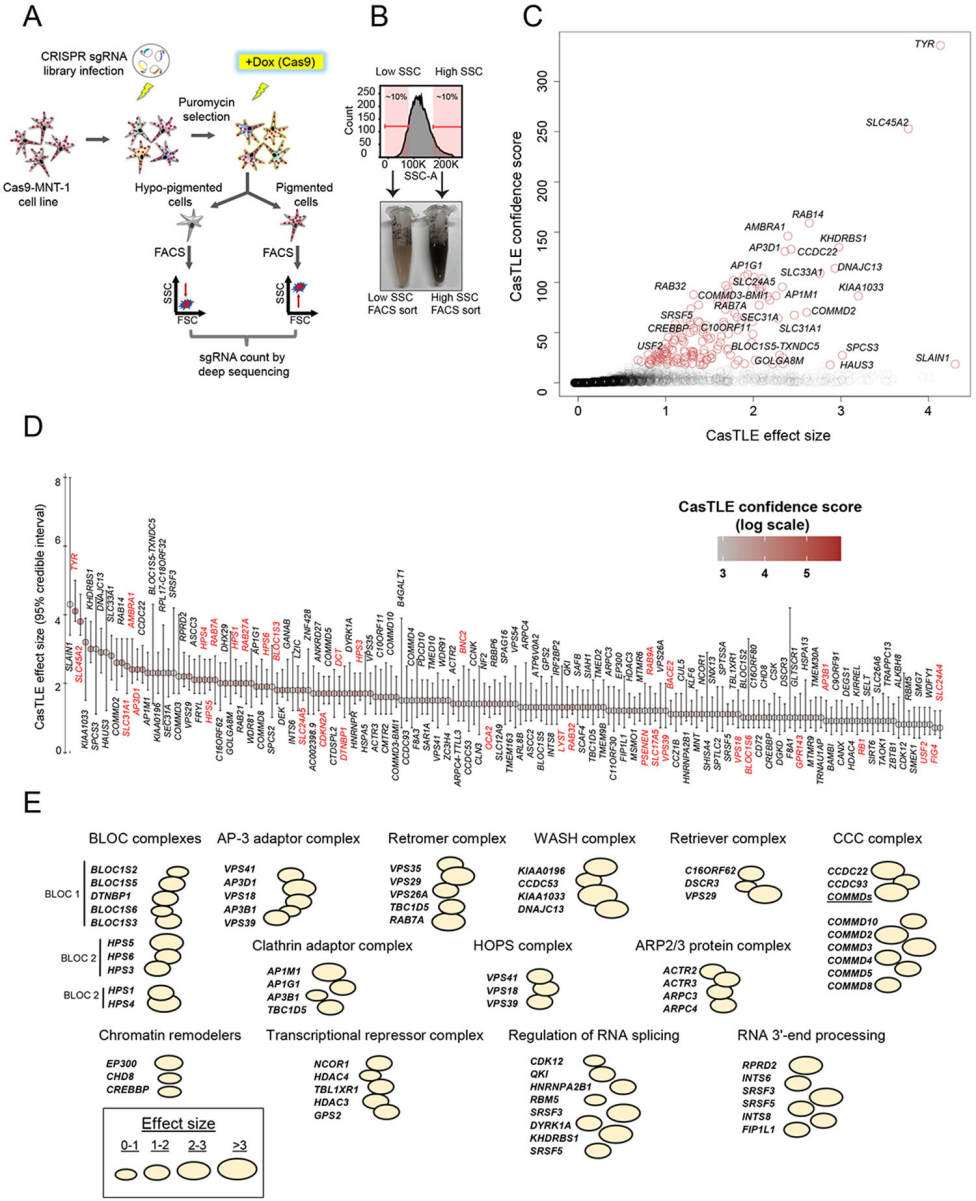
Genome-wide CRISPR-Cas9 screen for regulators of human melanogenesis. (**A**) Schematic of the screen design. (**B**) FACS cell sorting on low and high SSC enriches hypopigmented and hyperpigmented cells, respectively. (**C**) CasTLE likelihood ratio test analysis of two independent genome-wide pigmentation screens. Genes at <10% FDR cutoff are indicated with brown circles. (**D**) The maximum effect size (center value) estimated with CasTLE from two independent genome-wide pigmentation screens with 10 independent sgRNAs per gene. Bars, 95% confidence interval. Melanogenesis regulators as sorted by CasTLE effect size. The colors indicate CasTLE confidence score (log scale). Previously known pigmentation genes are high- lighted in red. (**E**) Classification of screen hits on the basis of biological functions and presence in common macromolecular complexes. Bubble size indicates CasTLE effect size for the respective gene in the screen. Bubbles touching each other indicates that the proteins have been reported to make a physical protein complex, with the name of the complex and/or associated molecular function indicated on top.

We reasoned that the close correlation between melanin content and SSC could be used as a paradigm for a CRISPR-Cas9 genetic screen, in which the loss of genes important for melanosome biogenesis and maturation would result in diminished SSC (Fig. 2A). We generated a clonal MNT-1 cell line that, when induced, expresses SpCas9 nuclease upon doxycycline treatment and infected it with a genome-wide lentiviral single guide RNA (sgRNA) library (20) (Cas9-MNT-1 cell line; fig. S2A). After puromycin selection to kill non-infected cells, expression of Cas9 was induced to carry out gene-editing, and after 2 weeks, cells from the top and bottom 10% of the SSC distribution were sorted with fluorescence- activated cell sorting (FACS) (Fig. 2A and B). As we expected, the low SSC fraction was enriched for hypopigmented cells relative to the high SSC fraction (Fig. 2B). Genomic DNA was subsequently isolated, and the frequencies of sgRNAs in both populations (low and high SSC) were measured with deep sequencing and analyzed by using the Cas9 high-throughput maximum likelihood estimator (CasTLE) algorithm, which provides a confidence score for the effect of each gene (21). Analysis of CasTLE scores in two independent biological replicates (i.e., independent sgRNA infections and FACS sorting after Cas9 induction) revealed good concordance (R^2^ = 0.59, P < 2.2 × 10^−16^) between the replicates (fig. S2B). We focused our subsequent analysis on genes with a positive CasTLE effect (those whose loss corresponded to a reduction in SSC) and which are therefore predicted to have a melanin-promoting function. Although our screen also identified genes with a negative CasTLE effect (e.g., those enriched in the high-SSC sorted fraction), we reasoned that gain of SSC may be more challenging to interpret because it can be affected by other factors that increase cellular granularity (22).

Our screen identified 169 putative melanin-promoting genes (i.e., genes whose deletion was associated with diminished SSC) in common between the two biological replicates at <10% false discovery rate (FDR) (Fig. 2, C and D, and table S1). We cross-referenced our 169 screen hits with a curated list of 650 pigmentation-associated genes (6). Notably, 34 of the 169 screen hits corresponded to known melanogenic genes (e.g., TYR, OCA2, SLC45A2, DCT, SLC24A5, HPS1, HPS3-6, LYST, AP3D1, and others, highlighted in red in Fig. 2D), whereas 135 (79.9%) screen hits represented previously unidentified candidates for regulation of melanogenesis.

At lower statistical confidence, between 10 to 20% FDR, the screen discovered an additional 75 hits (table S1), which included several known pigmentation genes (e.g., MYO5A, ATP7A, and RAB1A). This suggested that the 10% FDR cutoff we selected for our analysis is conservative and may miss additional genuine hits. Our screening paradigm had several limitations that precluded us from recovering all previously known pigmentation genes. First, it focused on measurement of melanin content of differentiated pigmented cells; therefore, genes that affect pigmentation by contributing to the developmental process of melanocyte lineage formation, but not to melanin content in mature melanocytes, would have been missed in our screen. Second, genes that are essential in a particular cell type are frequent dropouts in genetic screens (23-25). Given that key pigmentation-associated genes such as MITF, SOX10, and PAX3 are essential for melanocyte survival (26-29), this may potentially explain why these genes were not identified in our screen; future screens for genes essential in MNT-1 cells may help to clarify this. And third, our screen focused on genes with melanin-promoting function and therefore missed suppressors of melanogenesis, such as TPCN2 (30, 31) or *MFSD12* (8, 32).

Despite these noted limitations, our screen uncovered many genes that are known to regulate steps of melanosome maturation and whose functional loss leads to oculocutaneous albinisms (OCAs) and ocular albinism (OA) syndromes in humans, such as TYR (OCA1), OCA2, SLC45A2 (OCA4), SLC24A5 (OCA6), LRMDA (OCA7), DCT (OCA8), and GPR143 (OA). These results further highlight that our screening approach captures genes involved in melanosome biogenesis.

## Pigmentation screen hits regulate melanosome biogenesis and maturation through diverse biological mechanisms

Gene ontology analysis of screen hits revealed enrichment for melanosome organization, pigment granule organization, vesicle organization, and intracellular transport (fig. S3A and table S2). The screen hits were not only enriched for previously known melanosome biogenesis– or maturation-related factors such as BLOC (biogenesis of lysosomal organelles complex) and AP-3 adaptor complexes (4, 5), but also encompassed genes whose products form molecular complexes implicated in endosomal recycling [such as retromer, retriever, WASH, ARP2/3, and CCC (COMMD-CCDC22- CCDC93) complexes] (33-35) (Fig. 2E, fig. S3B and Table S2). These results emphasize the role of endosome recycling pathways in melanosome biogenesis and human pigmentation. Screen hits also included genes regulating chromatin modification and remodeling, as well as RNA splicing and processing, which suggests that both transcriptional and posttranscriptional modes of gene regulation contribute toward melanogenesis (Fig. 2E).

## 67.7 % of screen hits are more highly expressed in primary melanocytes from darkly pigmented individuals

**Fig. 3.**
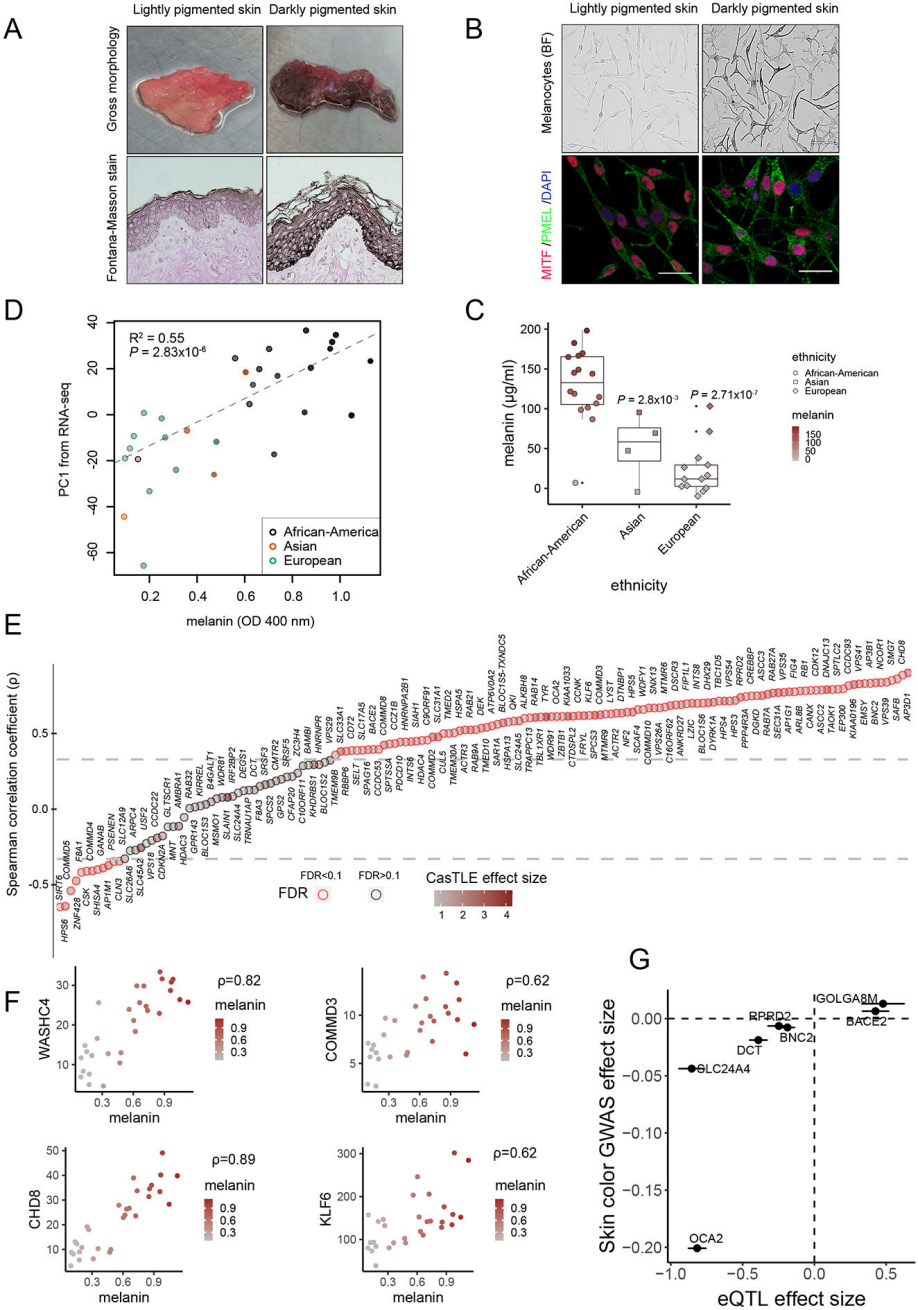
Pigmentation screen hits are differentially expressed in melanocytes of distinctly pigmented individuals. (**A**) Gross morphology of foreskin tissue obtained from diversely pigmented human donors. Fontana-Masson (FM) staining shows melanin pigment (black) in epidermis. (**B**) Brightfield (BF) and immuno- fluorescence (IF) images of melanocytes confirm differential pigment levels and presence of melanocyte markers MITF (red) and PMEL (green). DNA, blue. (**C**) Melanin quantification of melanocytes derived from diversely pigmented individuals. Box plots show median; whiskers are 1.5x IQR. Significance tested with ANOVA followed by two-sided Welch t-test with BH correction. (**D**) Correlation between RNA-seq gene expression profiles and melanin content of melanocytes. Plotted is melanin concentration (OD 400 nm) versus first principal component of the RNA-seq analysis from the same melanocytes. Ethnicity is indicated by the color of the outline of the plotting symbol. Fill color within each point represents measured melanin content. (**E**) Spearman’s r comparing relationship between TPM of candidate screen hits with melanin measurements in 30 melanocyte samples of diverse skin color. The intensity of brown color within each point indicates CasTLE effect size. Horizontal lines indicate (–0.33, 0.33) correlation coefficient cutoffs at <10% FDR. (**F**) Spearman’s ρ for selected CRISPR screen hits, with each point representing measurements from one human donor. Plotted is melanin OD at 400 nm (ordinate) versus RNA-seq expression level (TPM, abscissa). (**G**) Concordant effects of melanocyte eQTL and skin color GWAS for select melanin-promoting gene hits. For each of the indicated genes, the effect of a SNP’s alternate allele on that gene’s expression (defined as the slope of the eQTL; x axis) is plotted against the same allele’s effect on skin color from GWAS in white British individuals (*β* value from GWAS, where positive is associated with darker skin). Points and bars represent mean estimate ± standard error. Scale bars, (A) and (B), 25 μm.

We reasoned that because our screen has uncovered candidate melanin-promoting genes, at least some of these genes should be differentially expressed in melanocytes isolated from individuals with dark versus light skin color. These differences do not necessarily imply genetically encoded differences in expression, but rather that they may arise as a consequence of different rates of melanogenesis, in turn leading to a differential expression of melanogenesis-associated genes. To survey transcriptomes of diversely pigmented human melanocytes, we obtained foreskin tissues from 33 newborn males of diverse skin color and isolated primary melanocytes from them (Fig. 3A and table S3). Foreskin tissues were histologically analyzed for melanin presence in situ with Fontana-Masson staining, which confirmed the presence of differential melanin content in the epidermis of diversely pigmented individuals (Fig. 3A). Isolated melanocytes all expressed key lineage transcription factor MITF and melanosome specific marker PMEL but had distinct pigmentation levels (Fig. 3B). We spectrophotometrically quantified total melanin content of isolated melanocytes (Fig. 3C and fig. S1F). As we expected, melanin content correlated with the parent-reported donor ancestry, with melanocytes derived from African-American donors showing higher melanin content, followed by Asian and European donors, respectively (Fig. 3C and table S3).

Next, we performed RNA sequencing (RNA-seq) on isolated diversely pigmented melanocyte samples (n = 30) (table S4). Principal components analysis (PCA) of RNA-seq results demonstrated that gene-expression divergence correlated well with measured melanin content of the cells, even more so than reported ethnicity (Fig. 3D). We then calculated Spearman’s rank correlation coefficients (r) between expression (TPM, transcripts per kilobase million) of genes corresponding to our screen hits and melanin content [optical density (OD), 400 nm]. Out of the total 169 screen hits, 158 genes had measurable expression in all melanocytes and were therefore used for these calculations. We found that the expression of 107 hits (out of 158; 67.7%) was significantly positively correlated (q value < 0.1) with melanin, 12 genes were negatively correlated (q value < 0.1), and 39 were not significant (q value > 0.1) (Fig. 3, E and F, fig. S4, and table S5), demonstrating that the majority of screen hits are more highly expressed in darkly pigmented melanocytes. The key known skin variation genes picked up by our screen, such as OCA2 and SLC24A5, as well as genes encoding BLOC and AP complexes involved in melanogenesis, showed significant up-regulation in dark melanocytes (with a notable exception of BLOC-2 subunit HPS6, which showed negative correlation) (Fig. 3E, fig. S4, and table S5). Notably, endosomal recycling complexes identified in this study—including components of retromer, retriever, WASH, ARP2/3, and CCC complexes— were also differentially expressed between lightly and darkly pigmented melanocytes (figs. S4 and S5). At the gene regulatory level, genes that encode chromatin remodelers and modifiers (EP300, CHD8, and CREBBP), transcriptional repressor complex (NCOR1, HDAC4, and TBL1XR1), alternative mRNA splicing regulators (QKI, HNRNPA2B1, and DYRK1A) and RNA 3′-end–processing factors (INTS6, INTS8, and FIP1L1) were all significantly up-regulated in dark melanocytes (Fig. 3, E and F, and figs. S4 and S5). Altogether, these results further support the connection between identified screen hits and pigment levels in primary melanocytes isolated from human skin.

## Differences in expression of select screen hits may contribute to skin color variation in humans

As discussed previously, the differential expression of the majority of screen hits between darkly and lightly pigmented individuals is likely a secondary consequence of their involvement in sustaining a high rate of melanin synthesis, rather than being indicative of the role in driving skin color variation in humans. Nonetheless, given that one of the key mechanisms by which phenotypic variation arises is through gene regulatory divergence (36, 37), it is likely that for select loci identified in our screen, divergence in gene expression is indeed genetically encoded and may contribute to skin color variation. To provide a proof of principle for this hypothesis, we first asked whether the pigmentation screen hits are enriched for skin color heritability. We applied stratified linkage disequilibrium score regression (S-LDSC) (38) to assess skin color heritability enrichment in the vicinity (within 100 kb) of our screen hits by using publicly available summary statistics from a GWAS of individuals of white British ancestry in the UKBB. Skin color in this GWAS was represented as a self-reported, categorical trait (details of categories can be found in the supplementary materials, materials and methods), with negative GWAS effect sizes indicating a greater fraction of individuals self-reporting with lighter skin color categories. The 169 screen hits (at FDR < 10%) were significantly enriched for skin color heritability (fold- enrichment 9.61; standard error 3.80; P = 3.11 × 10^−2^) in comparison with all nonhits (FDR > 10%) (fold-enrichment 1.15; standard error 0.073; P = 3.37 × 10^−2^) (fig. S6A). Next, we collected lists of genes implicated by various GWASs, including (i) all genes annotated to pigmentation or skin color–associated single- nucleotide polymorphisms (SNPs) in the National Center for Biotechnology Information (NCBI)–European Bioinformatics Institute (EBI) GWAS catalog, representing a mix of ancestries (39); (ii) genes nearby lead SNPs associated with skin color variation in white individuals from the UKBB; and (iii) genes nearby lead SNPs associated with pigmentation variation in African individuals (8). Relative to all other genes assayed, screen hits were strongly enriched for pigmentation genes across the three datasets (table S6). For example, 8 of 169 hits lie closest to one of the top pigmentation-associated SNPs in Africans (8), compared with 322 of 20,044 nonhits (two-sided Fisher’s odds ratio 3.19; P = 5.14 × 10^−3^). Similar enrichments were observed for genes in the GWAS catalog (two-sided Fisher’s odds ratio 11.3; P = 6.13 × 10^−6^) or the UKBB (two-sided Fisher’s odds ratio 5.6; P = 2.75 × 10^−5^). Although some of the screen hits associated with GWAS peaks have known roles in pigmentation, others are newly identified. Of the screen hits in vicinity of the lead GWAS signals in the UKBB, three (AP1G1, SLC12A9, and SLAIN1) represent previously unknown associations with melanogenesis (Fig. S6B).

We further reasoned that if the expression levels of our screen hits causally affect pigmentation, then genetic variants that are quantitatively associated with increased expression (eQTLs) should also be associated with darker skin color in GWAS, and conversely, variants associated with decreased expression should be associated with lighter skin color. To this end, we identified the most significant and closest eQTL (with P value < 0.01) for each screen-hit gene by using a published melanocyte eQTL dataset (obtained mostly from European-ancestry skin samples) (40). We then correlated the eQTL effect of the alternate allele with the same allele’s effect on skin color in GWAS from white British individuals in the UKBB (also requiring GWAS P value < 0.01). These filtering steps left only seven screen hits with both eQTL and GWAS effect sizes, but notably, the direction of the eQTL and GWAS effects agreed for all seven of these genes (binomial P value = 0.01563). Specifically, the expression-increasing alleles for BACE2 and GOLGA8M are associated with darker skin color (positive on y axis means darker skin color) (Fig. 3G). GOLGA8M sits between the OCA2-HERC2 locus and APBA2 gene. A previous GWAS study (9) uncovered the association of noncoding variants in the intronic region of the APBA2 with human skin color. Although the authors suggested OCA2—located ~1 Mb away—as a candidate target gene regulated by these variants, the association of GOLGA8M with melanogenesis in our screen along with the identification of the concordant eQTL and GWAS signals at the locus suggests that GOLGA8M should also be considered as a potential target of the skin color–associated noncoding variants in the APBA2 introns. Conversely, expression-decreasing alleles for OCA2, BNC2, RPRD2, DCT, and SLC24A4 are associated with lighter skin color. These results indicate that variable expression of select screen hits may contribute to the skin color variation in humans.

## Select pigmentation screen hits display signatures of adaptive evolution in human populations

Skin color is thought to be under strong natural selection (3). To identify genes among our screen hits that might have undergone a recent adaptation for skin pigmentation variation among human populations, we used a tree-based statistic called population branch statistic (PBS) (41). In a three-population tree topology, PBS measures the change in allele frequency in the history of a target population since its divergence from the other two populations, with larger branch-specific PBS indicating population-specific, adaptively evolved SNPs. We computed genome-wide PBS per SNP using the African, European, and East Asian populations from the 1000 Genomes Reference Panel and created Manhattan plots of PBSs in the African and European target populations of this three-population tree (fig. S6, C and D).

Given that SNPs in the gene regulatory regions are a prime target for selection (42, 43), we included ± 100 Kb regions flanking the gene bodies of the screen hits in our analysis. For these loci (fig. S6, C and D, orange), we computed PBS statistics for the European, African, and East Asian populations of the 1000 Genomes Reference Panel (fig. S6, C and D, and tables S7 and S8). As shown in the Manhattan plot (fig. S6C) for the African population, several SNPs within these genic loci have PBS scores that are larger than the top 0.01% quantile of genome-wide PBS scores (orange dots above the dashed red line), which include OCA2, VPS39, CDKN2A, and BACE2 (fig. S6C and table S7). In the more relaxed setting, several melanin-promoting genic loci (i.e., COMMD3, SLC33A1, MTMR9, and TMED2) SNPs are larger than the top 0.1% quantile of genome-wide PBS scores (fig. S6C, orange dots above the blue dotted line, and table S7). Similarly, European PBS scores, which were also computed in the African–European–East Asian trio, showed SLC45A2, SLC24A5, OCA2, BNC2, LRMDA (44), and GOLGA8M loci SNPs with PBS scores larger than the top 0.01% quantile of genome-wide threshold (fig. S6D and table S8). To our knowledge, this is the first time that LRMDA (c10orf11/OCA7), a locus previously associated with eyebrow color in Europeans, has been implicated in adaptive evolution in a human population (44). At a relaxed threshold of 0.1% quantile (fig. S6D, blue dotted line, and table S8), we found additional SNPs within melanin-promoting loci with high PBS values, including ACTR3, TMEM163, HDAC4, RAB21, and NCOR1 loci. Our analysis reaffirms adaptive selective pressure on known pigmentation-associated genes such as SLC45A2 and SLC24A5 (45-47) and identifies variants near select candidate genes that are enriched for signatures of local adaptation. These newly identified variants will require further functional genomic analyses to pinpoint their cis- regulatory function and to confirm the target genes upon which they act.

If positive selection signatures observed for screen hits are because of selection on skin pigmentation, then alleles with increased frequency in a given population should be associated with pigmentation in a direction concordant with the overall change in pigmentation in that population relative to others. To test this hypothesis, we focused on SNPs with increased frequency in Europeans, owing to the availability of highly powered, publicly available summary statistics of skin color GWAS in white British individuals from the UKBB. In this GWAS, negative effect sizes for a given allele indicate a larger fraction of white British individuals self-reporting with lighter skin color categories, with the opposite for positive effect sizes. For SNPs within known pigmentation-associated screen hits, there was a significant, negative correlation between European PBS-score significance (the −log10 P value of the PBS score) and GWAS effect size (Spearman r = −0.42, P = 3.23 × 10^−8^) (fig. S7B). Alternatively, when we stratified variants associated with the loci on the basis of the significance of the PBS scores, it was apparent that alleles with the lower P value (<0.01) were preferentially associated with lighter self-reported skin color categories (as indicated by the negative GWAS effect size) compared with alleles with less significant (P value > 0.01) PBS scores (Wilcoxon rank- sum test P = 8.01 × 10^−5^) (fig. S7A). This indicates that alleles with increased frequency in Europeans are preferentially associated with lighter self-reported skin color categories. We observed analogous, although substantially weaker, effects when considering SNPs in screen hits with no previously known role in pigmentation both in correlational analysis (Spearman r = −0.13, P = 0.00016) (fig. S7C) and stratified analysis (Wilcoxon rank-sum test P = 5.4 × 10^−3^) (fig. S7A). This is to be expected, given that genes with strongest effect sizes on pigmentation in human populations have likely already been discovered. Nonetheless, our results demonstrate that both known and, to a lesser degree, newly identified loci harbor SNPs with some evidence for evolving under positive selection for skin pigmentation changes, which warrants their deeper exploration in future studies.

## Secondary validations confirm involvement of screen hits in different stages of melanosome biogenesis

Altogether, our results thus far uncover 135 new candidate melanin-promoting genes associated with diverse biological processes, demonstrate increased expression in dark-skin melanocytes for the majority of the screen hits, and suggest that some loci identified in our study may be involved in skin color variation and associated with evolutionary adaptations in human populations.

**Fig. 4.**
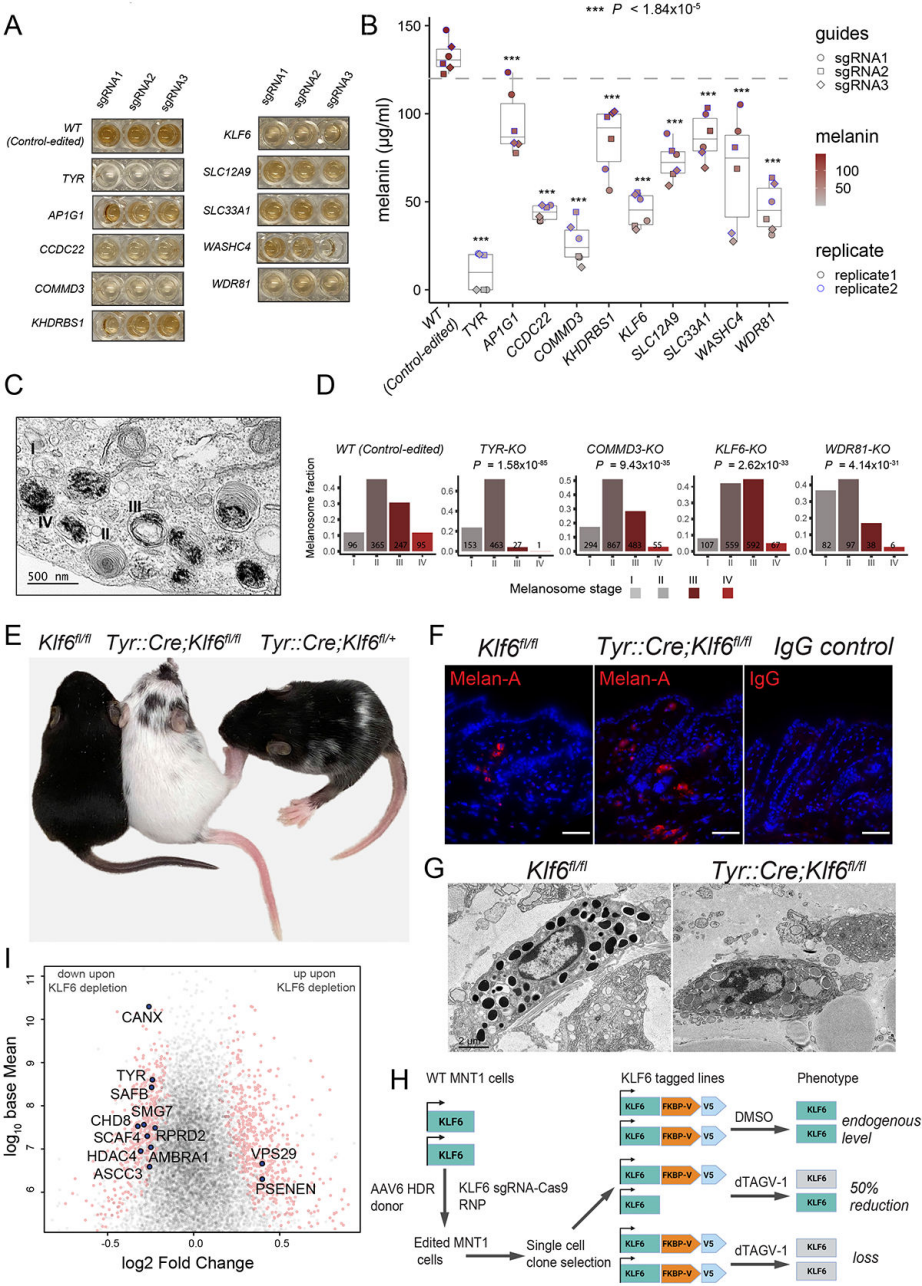
Novel pigmentation genes affect different aspects of melanosome biogenesis and maturation. (**A**) CRISPR- Cas9–based validation of select previously unidentified pigmentation screen hits along with negative (safe- targeting sgRNAs; wild type) and positive (TYR) controls. Cas9-MNT-1 cell lysates show gross changes in melanin levels upon deactivation of indicated genes using three different sgRNAs. (**B**) Boxplots show median melanin measurements for select gene knockouts and controls. P values relative to control-edited cells ranged from smallest 2.46 × 10^−20^ (for TYR-KO cells) to largest 1.84 × 10^−5^ (for AP1G1-KO cells). ***P < 1.84 × 10**−5**. (**C**) Representative TEM image showing melanosomes at stages I to IV of maturation. (**D**) Quantification of melanosomes at different stages of maturation among different indicated gene deactivations. Total counts are shown on each bar. P values from c-squared test are shown. (**E**) KLF6 regulates melanosome maturation, and its loss leads to severe pigmentation defects in vivo. P15 mice pups with homozygous deletion of Klf6 (TyrCre::Klf6^fl/fl^) display loss of melanin in hair coat color, toes, and tail. Mice with heterozygous deletion of Klf6 (TyrCre::Klf6^fl/+^) display diffuse dilution of toe, tail, and hair coat color, and variegated white patches compared with those of control animals with no Tyr::Cre expression (Klf6^fl/fl^). (**F**) Immunohistochemistry of mouse skin shows presence of Melan-A–positive melanocytes in Klf6 null (TyrCre::Klf6^fl/fl^) and control (Klf6^fl/fl^) animals. (**G**) TEM images of mouse skin showing black melanosomes within the melanocytes of control (Klf6^fl/fl^) animals compared with the presence of immature melanosomes in Klf6 null (TyrCre::Klf6^fl/fl^) mouse skin. (**H**) Schematic diagram of KLF6 endogenous tagging at C-terminus with FKBP12^F36V^ and V5 epitope. (I) RNA-seq volcano plot showing differentially expressed genes (highlighted in pink circles) in response to 24-hour dTAG^v^-1 treatment. Labeled genes (blue solid circles) are melanin-promoting genes discovered in CRISPR screen. Scale bars in (F), 25 μm; (C), 500 nm; and (G), 2 μm.

To further validate select hits representing a range of biological functions and screen-effect sizes, we introduced sgRNAs against AP1G1, CCDC22, COMMD3, KHDRBS1, KLF6, SLC12A9, SLC33A1, TYR, KIAA1033 (WASHC4), and WDR81 into the Cas9-MNT-1 cell line (Fig. 4, A and B, and fig. S8A). We conducted multiple gene- deactivation (knockout, KO) experiments using three distinct sgRNAs against each of the aforementioned genes, and the results confirmed their regulatory roles in human melanogenesis, as deactivating each gene significantly reduced pigmentation, albeit to varying degrees (Fig. 4, A and B). As we expected, the reduced pigmentation phenotype was accompanied by a diminished SSC (fig. S8B). Our validation experiments were done on bulk cell populations, which likely contained a mixture of heterozygous, homozygous, and unedited cells. Thus, melanin measurements underestimate the loss of gene pigmentation phenotype expected from a complete (homozygous) loss of gene function.

To gain insights into how select hits may affect melanosome biogenesis, we performed TEM imaging on KLF6-, COMMD3-, and WDR81- KO cells and quantified melanosomes at various stages of maturation in comparison to wild-type (control-edited) and TYR-KO cells (Fig. 4, C and D, and fig. S8C). We found that the loss of KLF6 was associated with a significantly reduced proportion of stage IV melanosomes (one-sided two-sample test of proportions, P = 2.62 × 10^−33^) (Fig. 4D and fig. S8C), which explains the reduced pigmentation of the knock- out cells (Fig. 4, A and B) and suggests that this transcription factor regulates expression of genes required for the later stages of melanosome maturation. Deletion of COMMD3 led to a significant increase in stage I and II non- melanized melanosomes (one-sided two-sample test of proportions, P = 9.43 × 10^−35^) in which fibrillar structure was visible, but transition to stage III or IV appeared to be defective (Fig. 4D and fig. S8C), which was reminiscent of the TYR-KO phenotype (figs. S1I and S8C). We found that WDR81-KO cells had a significantly higher proportion of stage I melanosomes compared with controls (one-sided two-sample test of proportions, P = 4.14 × 10^−31^), which was evidenced by the presence of multiple vacuoles or vesicles (Fig. 4D and fig. S8C). Because stage I melanosomes are derived from vacuolar domains of the early endosomal compartments (5, 17, 48), WDR81 KO likely affects the early endosomal compartments. In agreement, a recent study showed that loss of WDR81 caused an increase in endosomal phosphatidylinositol 3-phosphate (PtdIns3P), leading to the enlargement of early endosomes (49). Thus, our screen hits are involved in the regulation of melanosome biogenesis and maturation at different stages, which suggests diverse biological mechanisms.

## KLF6 regulates melanosome maturation in vivo

To gain deeper insights into the mechanisms by which these novel genes regulate pigmentation, we further focused on two hits that were involved in distinct molecular processes: transcription factor KLF6 and endosomal recycling protein COMMD3. First, we conditionally deleted one or both copies of the Klf6 gene in the melanocytic lineage by crossing Klf6^fl/fl^ (50) mice with Tyr::Cre mice that express Cre recombinase under melanocyte-specific Tyr gene promoter (51). Given that the Tyr::Cre transgene was located on the X chromosome (51), which was subject to random X inactivation in females, we focused our analyses only on male progenies. All animals with homozygous loss of Klf6 (TyrCre::Klf6^fl/fl^) displayed severe loss of melanin in hair coat color and complete loss of melanin in toe and tail, both of which are devoid of any hair and are equivalent to interfollicular epidermis in humans (Fig. 4E, fig. S9A, and table S9). By comparison, heterozygous melanocyte-specific loss of Klf6 (TyrCre::Klf6^fl/+^) resulted in partial loss of melanin in tail and toe, diffuse dilution of coat color, and variegated loss of melanin visible as white coat patches in all animals (Fig. 4E, fig. S9A, and table S9). These results indicate a strong and dosage-dependent effect of KLF6 on pigmentation with complete penetrance (table S9). To assess whether this pigmentation phenotype results from defective melanosome maturation or the loss of melanocytic lineage, we performed skin histology and electron microscopy studies (Fig. 4, F and G, and fig. S9B). The average number of melanocytes (per unit area) in TyrCre::Klf6^fl/fl^ animals were similar to that of Klf6^fl/fl^ animals, as ascertained by counting Melan-A–positive melanocytes [53.05 ± 4.9 (SEM) melanocytes per square milimeter in TyrCre::Klf6^fl/fl^ animals versus 53.45 ± 5 (SEM) melanocytes per square milimeter in Klf6^fl/fl^ animals; P = 0.96)] (Fig. 4F and table S10). These results confirmed that loss of Klf6 does not affect development and survival of melanocytes (Fig. 4F and table S10). TEM analyses showed the presence of amelanotic melanosomes within TyrCre::Klf6^fl/fl^ melanocytes, confirming that KLF6 regulates later stages of melanosome maturation, which is in agreement with our in vitro KLF6-KO TEM analysis (Fig. 4G).

Given that KLF6 is a transcription factor, we proceeded to examine effects of its loss on gene expression using human MNT-1 cells. To this end, we endogenously tagged one or both alleles of KLF6 at the C-terminus with the FKBP12^F36V^ and V5 tags, which allow for rapid degradation of KLF6 in response to treatment with degradation tag (dTAG) molecules and protein detection with immunoblotting, respectively (Fig. 4H and fig. S9, C to F) (52). Because different dTAG degrader molecules may have variable protein degradation responses (52), we tested two commonly used dTAG molecules—dTAG-13 and dTAG^v^-1—and found dTAG^v^-1 more effective in KLF6 degradation in MNT-1 cells (fig. S9F). Therefore, we used dTAG^v^-1 for all subsequent KLF6 depletion experiments. Next, we depleted KLF6 in both homozygous and heterozygous MNT-1 cells and examined the effects of KLF6 loss on gene expression after 24 hours of dTAG^v^-1 treatment. Combined RNA-seq analyses of homozygous and heterozygous cell lines treated with either dTAG^v^-1 and dimethylsulfoxide (DMSO, vehicle control) revealed that more than 900 genes (410 down-regulated and 576 up-regulated) were differentially expressed in response to KLF6 depletion (Fig. 4I). Among 169 melanin-promoting genes uncovered through our CRISPR screen, the expression of 12 genes was significantly affected by the loss of KLF6 (Fig. 4I). Notably, 10 of these candidate genes (TYR, AMBRA1, CHD8, HDAC4, ASCC3, SAFB, CANX, SMG7, RPRD2, and SCAF4), including that of key melanogenic enzyme tyrosinase, were down-regulated in response to KLF6 depletion. This is in agreement with both their positive regulation by KLF6 and the promelanocytic role of KLF6 uncovered in our studies (Fig. 4I). Taken together, our results uncover a role for KLF6 in melanosome maturation and pigmentation and pinpoint candidate transcriptional targets that may mediate these functions.

## Endosomal trafficking protein COMMD3 modulates melanosomal pH

**Fig. 5.**
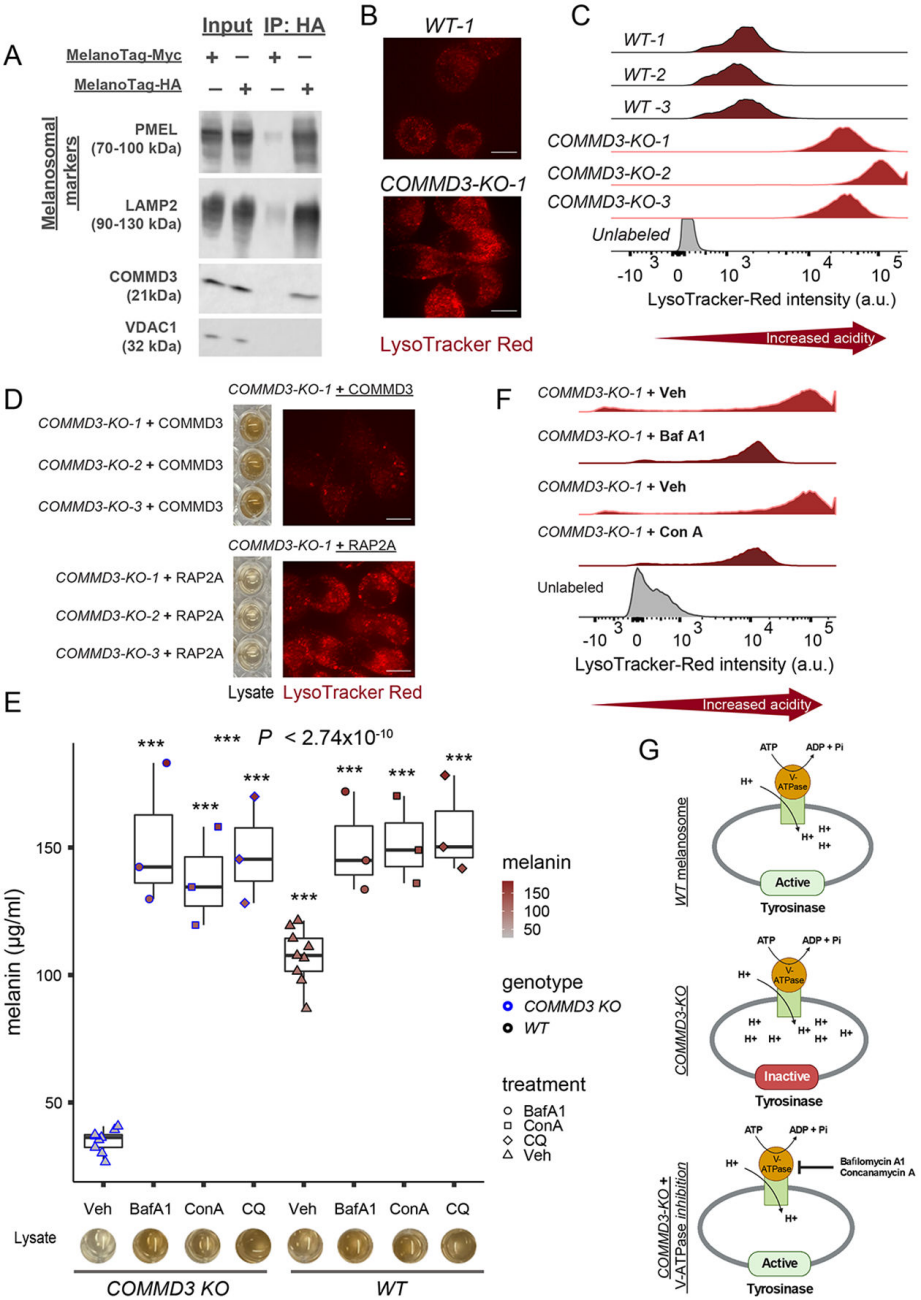
COMMD3 is enriched in melanosomes and regulates melanosomal pH. (**A**) COMMD3 is enriched in purified melanosomes. Immunoblots showing whole-cell lysates (input), con- trol (MNT-1 cells expressing a Myc- MelanoTag), and purified melanosome (MNT-1 cells expressing HA-MelanoTag) immunoprecipitates (IP). Melanosomal markers PMEL and LAMP2 are enriched in purified melanosomes in comparison with mitochondrial marker VDAC1. (**B**) COMMD3-KO cells display high acidity (low pH) of endosomal organ- elles (e.g., melanosomes) as confirmed with live cell imaging using LysoTracker Red dye (n = 3 clones). (**C**) Flow cytometry histograms showing increased LysoTracker Red fluorescence intensity in COMMD3-KO clones compared with wild-type (control-edited) clonal cells (n = 3). (**D**) COMMD3, but not RAP2A (32) (an unrelated and constitutively expressed protein), overexpression in COMMD3-KO cells rescues both melanin production and reduced pH (n = 3). (**E**) COMMD3-KO phenotype is rescued by neutralizing melanosomal pH. V-ATPase proton pump inhibitors bafilomycin A1 (BafA1, 0.1 mM), concanamycin A (ConA, 0.1 mM), and chloroquine (CQ, 50 mM) raise the pH of melanosomes in comparison with vehicle (Veh, DMSO and H2O) controls and restore melanin production as shown with melanin quantification and cell lysates (n = 3). Significance tested by ANOVA and two-sided Welch t-test. ***, P < 2.74 × 10^−10^ (for all groups, relative to vehicle group). (**F**) Flow cytometry histograms showing decreased fluorescence intensity of LysoTracker Red in COMMD3-KO cells treated with BafA1 (0.1 mM) and ConA (0.1 mM) compared with vehicle control (Veh) treated cells (n=3). (**G**) Schematic diagram of putative COMMD3 function in melanosomes. COMMD3-KO cells fail to neutralize maturing stage III and IV melanosomes, affecting TYR enzymatic activity. Neutralizing (i.e., raising the pH) melanosomes by blocking V-ATPase proton pump restores TYR activity and melanogenesis. Scale bars in (B) and (D), 25 μm.

We next focused on another hit from our screen: COMMD3, which belongs to the COMMD (copper metabolism MURR1 or COMM domain) family of proteins, comprising of 10 members (33, 34). COMMD proteins homo- or hetero- dimerize and associate with CCDC22 and CCDC93 proteins to form the CCC complex, which is involved in endosomal receptor trafficking (33, 34). CCDC22, CCDC93, and multiple COMMD genes (COMMD2, COMMD3, COMMD4, COMMD5, COMMD8, and COMMD10) of the CCC complex were identified as hits in our screen (Fig. 2E). Notably, COMMD1, a CCC complex component, has been implicated in the recycling of copper-transporting adenosine triphosphatase (ATPase) 1 (ATP7A) (33, 34). ATP7A regulates pigmentation by transporting copper to melanosomes, because copper binding to the TYR apoenzyme is essential for its holoenzyme activity (53). Contrary to our expectations, however, we found that COMMD3 KO does not affect the localization of ATP7A to melanosomes (fig. S10, A and B). We also did not detect any measurable effects of COMMD3 KO on TYR levels (fig. S10, C and D). Nonetheless, COMMD3 was enriched within the melanosomal fraction, as determined with melanosome immunoprecipitation (melano-IP) (32), which suggests that it may regulate pig- mentation by functioning in melanosomes or by promoting receptor trafficking to melanosomes (Fig. 5A).

An important regulator of TYR activity and pigmentation is melanosomal pH (5). Stage I and II melanosomes are characterized by an acidic environment, but stage III and IV melanosomes require neutral pH for optimal TYR enzymatic activity (5). To investigate the pH of melanosomes, we used lysoTracker Red DND-99, a fluorescent acidotropic pH indicator probe that stains all endosome-derived acidic organelles such as melanosomes and lysosomes. Notably, COMMD3 KO resulted in significantly higher acidity (low pH) of endosomal compartments including melanosomes, which was confirmed by live imaging and flow cytometry with LysoTracker Red dye (Fig. 5, B and C). Notably, costaining with TYR and lysotracker Red dye confirmed that TYR-positive melanosomes of COMMD3-KO cells were indeed highly acidic compared with wild-type (control-edited) cells (fig. S10, E and F). Reintroduction of COMMD3—but not of the unrelated and constitutively expressed protein RAP2A (32) —to COMMD3-KO cells restored both pH and melanosomal melanin production, which indicated specificity of the observed effects (Fig. 5D and fig. S10, G to I). We further tested whether increasing (i.e., neutralizing) melanosomal pH with alternative approaches can rescue the COMMD3-KO phenotype and restore melanin production. To this end, we treated COMMD3-KO cells with bafilomycin A1 (BafA1) and concanamycin A (ConA), which bind at distinct sites of the vacuolar-type H^+^-ATPase (V-ATPase) proton pump and selectively inhibit V-ATPase responsible for maintaining the acidity of endosomal compartments, including melanosomes (54). Both BafA1 (0.1 mm) and ConA (0.1 mm) raised the pH of endosomal compartments and completely restored melanin production in COMMD3-KO cells to a level similar to that of control cells (Fig. 5, E and F). In addition, treatment of COMMD3-KO cells with 50 mm chloroquine (CQ), which acts as a weak base and neutralizes luminal pH by accumulating within the endosomal organelles (55), also restored melanin production (Fig. 5E) (55). Thus, by restoring neutral melanosomal pH in a V-ATPase–dependent (BafA1 and ConA) and -independent (CQ) manner, we were able to completely restore melanosome maturation and melanin production in COMMD3-KO cells. These results strongly suggest that COMMD3- KO melanosomes contain all the required com- ponents for melanin production except for neutral melanosomal pH, which is essential for optimal TYR enzyme activity (Fig. 5G) (5). Thus, a plausible mechanism for COMMD3’s role in pigmentation is the regulation of melanosomal localization of one or more transporters responsible for modulating luminal pH.

## Discussion

We have demonstrated that changes in melanosomal composition can be robustly quantified by measuring the side-scattering property of pigment cells. By exploiting this relation- ship, we directly surveyed genes involved in melanosomal biogenesis and maturation without being confounded by the indirect factors influencing pigmentation through effects on melanocyte development, survival, migration, and other processes. The majority of previously unidentified melanogenesis genes uncovered in our study are more highly expressed in primary melanocytes of darkly pigmented individuals, which correlates well with their melanin-promoting function. Although it should be noted that our screen hits do not represent an exhaustive list of pigmentation genes, they nonetheless include 135 newly identified candidate regulators of melanogenesis involved in diverse molecular functions such as transcriptional and posttranscriptional gene regulation, vesicle organization, and intracellular transport, the latter including several molecular complexes involved in endosomal recycling. Although the key role of early endosomes in melanogenesis is well established, our work implicates new cargo recycling pathways in melanosome function. We showed that COMMD3, a component of the endosomal trafficking CCC complex, is enriched in melanosomes and regulates their luminal pH. Moreover, our work uncovered a new role for transcription factor KLF6 in regulating melanosome maturation and skin pig- mentation in vivo. Prior studies have reported that loss of KLF6 in collagen-rich environments leads to increased melanoma proliferation, which is consistent with the role of KLF6 as a tumor suppressor in multiple cancers (56-61). In light of our findings, it would be informative to examine the role of KLF6 inactivation in melanoma initiation and how the extracellular matrix could modulate this process. Furthermore, these and other hits from our screen merit further exploration in the context of other diseases, because aberrant melanin production is associated not only with skin pigmentation disorders but also with Parkinson’s disease and auditory disorders, which have been linked to loss of neuromelanin in substantia nigra and cochlear melanin in the inner ear, respectively (62, 63). Furthermore, select loci identified in our screen show association with skin color variation and evidence of recent adaptations in human populations. Therefore, in addition to the aforementioned insights into melanosome biology and disease, results presented here will provide a rich resource for further studies of the genetic architecture of skin color diversity in humans.

## Methods summary

To establish whether melanosome maturation can be monitored by measuring changes in SSC, we used two models of human melanogenesis: human pluripotent stem cell (PSC)– derived melanocytes and MNT-1 melanoma cells. We measured SSC and melanin content of human PSC–derived melanoblasts as they matured into melanocytes and conducted CRISPR deletion of TYR and rescue experiments in MNT-1 cells. Both orthogonal set-ups of experiments showed a strong positive correlation between melanin content and SSC. To perform the genome-wide CRISPR screen, we engineered a clonal MNT-1 cell line to express SpCas9 nuclease in a doxycycline-regulatable manner (Cas9-MNT-1 cell line). We infected the Cas9-MNT-1 cell line with a genome-wide lentiviral sgRNA library (with 10 targeting guides per gene) (20), so that each cell expressed a single sgRNA. In addition, the library also included negative control sgRNAs such as nontargeting guides (i.e., no binding sites in the genome) and safe-targeting control guides that targeted genomic loci with no annotated function (20).

## Supplementary Material

Table S1

Table S2

Table S3

Table S4

Table S5

Table S6

Table S7

Table S8

Table S9

Table S10

Supplementary Material

checklist
