# Supplementary material for: A genome-wide genetic screen uncovers determinants of human pigmentation
Source: Science. Author manuscript; Available in PMC 2024 Feb 28. (PMC10901463; doi:10.1126/science.ade6289)
Supplement: Supplementary Material [file NIHMS1964433-supplement-Supplementary_Material.pdf]

Science AAAS

Supplementary Materials for

A genome-wide genetic screen uncovers determinants of human pigmentation

Vivek K. Bajpai *et al*.

Corresponding author: Joanna Wysocka, wysocka@stanford.edu

Science **381**, eade6289 (2023)
DOI: 10.1126/science.ade6289

The PDF file includes:

Materials and Methods
Figs. S1 to S10
References

Other Supplementary Material for this manuscript includes the following:

Tables S1 to 10
MDAR Reproducibility Checklist

Materials and Methods

Cell culture

All cells were routinely tested for mycoplasma contamination using MycoAlert detection kit (Lonza, Cat# LT07-318).

Pluripotent stem cells (PSC) culture

The SOX10::GFP reporter H9 human embryonic stem cells (hESC) were initially cultured on mouse embryonic feeder cells as described previously (*65*) and gradually adapted to feeder-free culture. H9 hESC SOX10::GFP reporter line and H20961 human induced pluripotent stem cells (iPSCs) were cultured in feeder-free, serum-free medium mTESR-1 (Stem Cell technologies, Cat# 85850). Both pluripotent stem cells (PSC) i.e. H9-hESC SOX10::GFP reporter and H20961 iPSC were passaged ~1:6 every 4-5 days by incubation in ReLeSR (StemCell technologies, Cat# 05872) for 1 min at room temperature. The ReLeSR was then aspirated and the culture plates were incubated for 6-7 mins at 37ºC. mTESR-1 was added to the cells and the plates were gently tapped to detach the cells, which were triturated and re-plated on tissue culture dishes coated overnight with growth-factor-reduced Matrigel (Corning, Cat# 356231).

PSC differentiation into melanocytes

The SOX10::GFP reporter H9 hESC and H20961 iPSCs were differentiated into melanocytes following a previously published protocol with some modifications (14). Cells were plated on Matrigel coated plates in mTESR-1 medium and when colonies reached 70% confluency, the medium was changed to differentiation medium composed of KSR medium (1x knockout DMEM (Gibco, Cat# 10829018), 15% knockout serum replacer (Gibco, Cat# 10828028), 1x Antibiotic-Antimycotic (Gibco, Cat# 15240062) 1x MEM Non-Essential Amino Acids Solution (Gibco, Cat# 11140076), 1x Glutamax (Life Technologies, Cat# 35050061), 55 mM 2-mercaptoethanol (Gibco, Cat# 21985023) containing 10 µM SB431542 (Selleck Chemicals, Cat# S1067) and 500nM LDN193189 (Selleck Chemicals, Cat# S2618) for 48 hours with medium change every 24 hours. After 48 hours, in the above medium, 3 µM CHIR99021 (Selleck Chemicals, Cat # S2924) was added. After 72 hours, cells were fed with KSR media containing 10 µM SB431542 and 3 µM CHIR99021. On day 4 (after 96 hours), cells were fed with 75% KSR medium, 25% N2 medium plus 3 µM CHIR99021. The N2 medium is comprised of: 1x DMEM-F12 (Gibco, Cat# 11320033),1x N2 NeuroPlex supplement (Gemini Bio, Cat# 400-163), 5.76 x 10^{-5} mg/ml progesterone (Sigma, Cat# P7556), 1.55 mg/ml D-glucose (Sigma, Cat# G7021), 1x 55 mM 2-mercaptoethanol, 1x antibiotic-antimycotic. On day 6, cells were fed with 50% KSR, 50% N2 media containing 3 µM CHIR99021 plus 25 ng/ml BMP4 (BioVision, Cat# 4578) and 100 nM endothelin-3 (Alfa Aesar, Cat# J66312). On day 8, media was switched to 25% KSR media, 75% N2 media plus 3 µM CHIR99021, 25 ng/ml BMP4, and 100 nM EDN3. On day 10, cells were fed with 100% N2 media containing 3 µM CHIR99021, 25 ng/ml BMP4, and 100 nM EDN3. On day 11, cells were treated with Accutase (Sigma, Cat# A6964) for 30 mins and washed twice with neurobasal medium (Life Technologies, Cat# 21103049), counted, resuspended at a concentration of 2x10^6 cells/ml and plated on poly-L-ornithine (Sigma, Cat# P4638), laminin (Life Technologies, Cat# 23017015), and fibronectin (Fisher Scientific, Cat#

FC01010MG) coated dishes as 20 µl volume droplets. Droplets were incubated at 37°C for 20 mins followed by the addition of MEL medium (50% neurobasal, 30% low glucose DMEM (Life Technologies, Cat# 11885084), 20% MCDB201 (Sigma, Cat# M6770), 1x antibiotic-antimycotic, 1x Glutamax, 1x ITS supplement (Corning, Cat# 354352), 100uM ascorbic acid (Sigma, A4403), 50ng/ml cholera toxin (Sigma, Cat# C8052), 50ng/ml Stem Cell Factor (Fisher Scientific, Cat# 50276388), 50nM dexamethasone (Sigma, Cat# D4902), 100nM EDN3, 2% Gem21 Neuroplex (Gemini Bio, Cat# 400-160), 3 µM CHIR99021, 4ng/ml FGF2 (PeproTech, Cat# 100-18B), 500um dbcAMP (Sigma, Cat# D0627), 25ng/ml BMP4. MEL medium was changed every 3 days and cells were passed every 5-6 days. Cells started showing pigmentation past day 25 of differentiation. As the cells differentiated from melanoblasts to the melanocytes state, cells were analyzed for side scatter (SSC) changes by FACS, as well as melanin quantification by measuring OD at 400 nm and comparing against a standard curve generated using synthetic melanin.

Primary human melanocytes culture

Foreskin tissues were procured from 1-2 day-old newborn males of diverse ethnicity following Institutional IRB protocol guidelines. Skin tissues were processed as described previously (*66*). Briefly, tissues were washed 3 times with 1x PBS (Gibco, Cat# 14040216), cut into pieces (~5mm×5mm). A piece was fixed in 10% neutral buffered formalin and provided to the Stanford Pathology Core for Fontana-Masson staining. The rest of the tissue was enzymatically digested with dispase I (Zen-Bio, Cat# DISP1) for 15-20 hours at 4˚C. Afterwards, the epidermis was peeled from the dermis manually using fine forceps. The epidermis was treated with trypsin-EDTA (Corning, Cat# 25053CI) for 15-20 mins at 37˚C with intermittent trituration. The cell suspension was neutralized using soy trypsin neutralizing solution (Life Technologies), filtered through a 70 µm cell strainer (ThermoFisher Scientific, Cat# 22363548), centrifuged at 200g for 5 min, and cultured in the presence of serum-free medium 254 (ThermoFisher Scientific, Cat# M254500) supplemented with 1x human melanocyte growth supplement (HMGS) (ThermoFisher Scientific, Cat# S0025) for 72 hours without medium change. Afterwards, fresh medium was replenished every other day.

Melanoma cell line culture

MNT-1 cells were cultured in high glucose DMEM (Life Technologies, Cat# 11995040) supplemented with 10% fetal bovine serum (Life Technologies, Cat# 16000069), 1x antibiotic-antimycotic and 1x Glutamax.

Plasmids, SpCas9 expression, gene knockout and rescue cell lines generation

Pb-TRE3G-SpCas9-IRES-Blast (Addgene# 195506), a piggyBac doxycycline inducible SpCas9 expression vector (see image below), initially generated in the Wysocka laboratory, was used in this study.

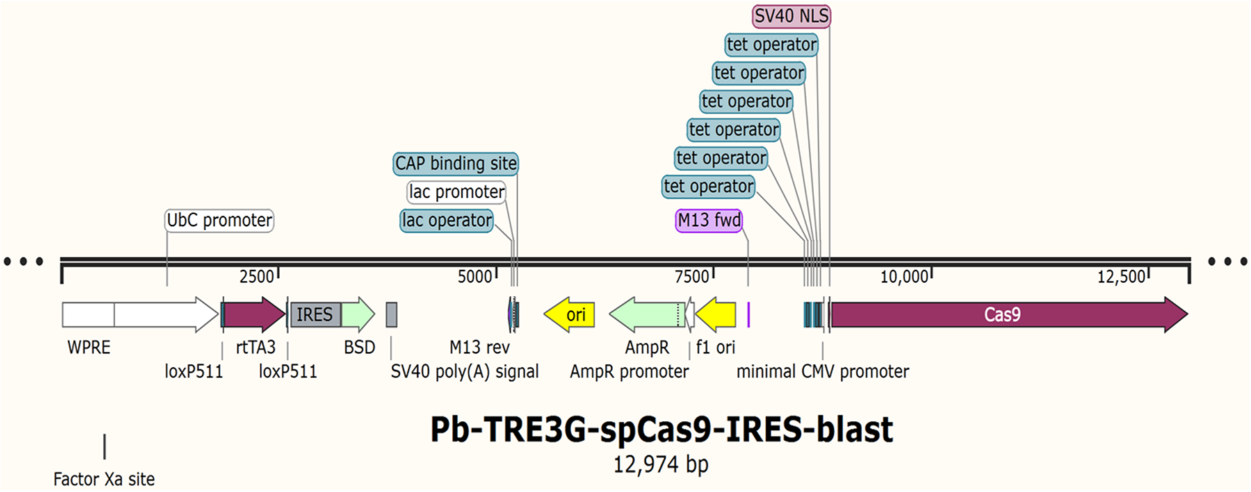

Map of Pb-TRE3G-SpCas9-IRES-blast plasmid

This plasmid efficiently expressed SpCas9 under a doxycycline inducible promoter and contained a blasticidin selection cassette. The plasmid was subsequently used for creating the Cas9-MNT-1 cell line that was used for both genome-wide screening and screen hit validation experiments. To generate the Cas9-MNT-1 cell line, MNT-1 cells were transfected with Pb-TRE3G-SpCas9-IRES-Blast and piggyBac transposase (PB210PA-1, System Biosciences) in a 1:3 ratio using Lipofectamine 2000 (Life Technologies, Cat# 11668019) following the manufacturer's instructions. Cells were selected with 5µg/ml blasticidin (InvivoGen, Cat# ant-bl-1) treatment for a week. Afterwards, single cells were plated to generate a clonal cell line and efficient induction of SpCas9 nuclease from the clonal cell line was confirmed by western blot. In the screen hit validation experiment, the Pb459-U6-sgRNA-EF1a-PuroR vector (Addgene # 195507) used for sgRNA expression in the Cas9-MNT-1 cells was designed in two steps. First, the lentiviral backbone of the pMCB320 vector was replaced with the piggyBac backbone from the PB-TRE-dCas9-VPR (Addgene, 63800) vector using EcoRI (NEB) and XbaI (NEB) restriction enzymes. Next, PuroR-T2A-mCherry was replaced with the PuroR cassette using NheI (NEB) and EcoRI (NEB). Pairs of oligonucleotides carrying sgRNA sequences (see table below) and the BlpI (NEB) and BstXI (NEB) overhangs were phosphorylated and annealed. The annealed oligos were subsequently ligated into the BlpI and BstXI digested, and gel purified, Pb459-U6-sgRNA-EF1a-PuroR backbone.

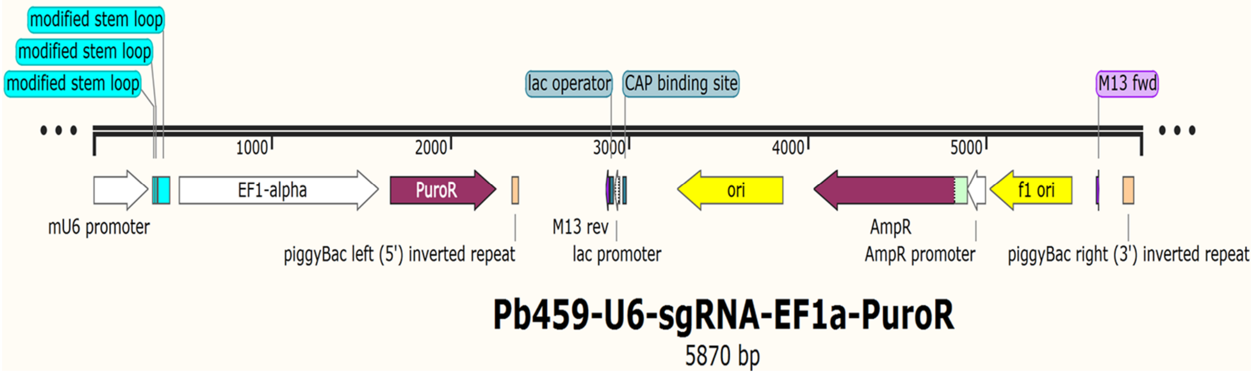

Map of Pb459-U6-sgRNA-EF1a_PuroR plasmid

Table sgRNA sequences used for knockout experiments.

**Gene**	**sgRNA sequence**
AP1-G1	GCTCCCGCAATCTGATGG
AP1-G2	GGGTTCGGGCTGTCCGGA
AP1-G3	GACAATACATACCGATGT
CCDC22-G1	GCAGGGTTGATCACACGC
CCDC22-G2	GTCTGCCCGGTTCCGCC
CCDC22-G3	GGGCTATCCCTTGGAGCT
COMMD3-G1	GCGGGGATCCGCCAGCATC
COMMD3-G2	GTACATCCTATGAAGTTGAT
COMMD3-G3	GATCTCTCCCTCATATAA
Control-G1	GTCATACATGTATTTGGA
Control-G2	GACAAGAAAAACCACTTA
Control-G3	GATACTAATCAATAAGTGGG
KHDRBS1-G1	GGCGTCTGACGCACCGAG
KHDRBS1-G2	GGGTCCCGACGCGACAGT
KHDRBS1-G3	GGGCCGTAGCGGCTCCA
KLF6-G1	GGAGGTAAACTTGGCCGT
KLF6-G2	GCCGGTCTCGTGCACGATC
KLF6-G3	GCTGTCAAATTTGATTTCTG
SLC12A9-G1	GCGAGAGCTCACCTCTGC
SLC12A9-G2	GGCGACGATCGTGCCCAG
SLC12A9-G3	GCAGGACATAGACGAAGA
SLC33A1-G1	GCCGGCTGCTGTCCTTGT
SLC33A1-G2	GGACTCAGCGGGCCGGGA
SLC33A1-G3	GCAAAATGCTGCTTAGTT
TYR-G1	ATTGTCTGTAGCCGATTGG
TYR-G2	GCTGTCCACCGTGGAGCG
TYR-G3	GTGCTCTGGCAACTTCAT
TYR-G4	GAGGAGACACAGGCTCTA
WASHC4 (KIAA1033)-G1	GGTGATTGCCAAATTCAAAT
WASHC4 (KIAA1033)-G2	GCGAGCCGTCGTCAACG
WASHC4 (KIAA1033)-G3	GTACGAATGATAAGATCTGG
WDR81-G1	GCAGGCAGATGAGGGCGATG
WDR81-G2	GTCGCAACCCTGCCAGCG
WDR81-G3	GACGTCAGCAGGCGGAGC

After cloning the Pb459-U6-sgRNA-EF1a-PuroR vector expressing unique sgRNAs, Cas9-MNT-1 cells were transfected with Pb459-U6-sgRNA-EF1a-PuroR and piggyBac transposase (PB210PA-1, System Biosciences) in a 1:3 ratio using Lipofectamine 2000 following manufacturer's instructions. Transfected cells were selected with 1μg/ml puromycin treatment for 4-6 days. Afterwards, cells were treated with 2μg/ml doxycycline (Sigma, Cat# D5207) for 2 weeks followed by cell lysis, melanin quantification, and FACS analysis on FACSAria (BD)/

CytoFLEX (BD) for SSC measurements. FACS analyses were done using FlowJo software (BD) and flowCore() package in R.

To generate the *TYR* expression plasmid used in the tyrosinase rescue studies, a wild copy of the *TYR* gene was PCR amplified from the pEGFP-TYR plasmid (Addgene # 32781) with NheI and AgeI overhangs. The amplicon was then digested and ligated to the already digested and gel purified PB-TRE-dCas9-VPR plasmid (Addgene, 63800) backbone. The resulting vector Pb-TRE3G-TYR-HygR (Addgene # 195508) (see image below) contained the TYR cassette under the doxycycline inducible TER3G promoter and also contained a hygromycin selection cassette.

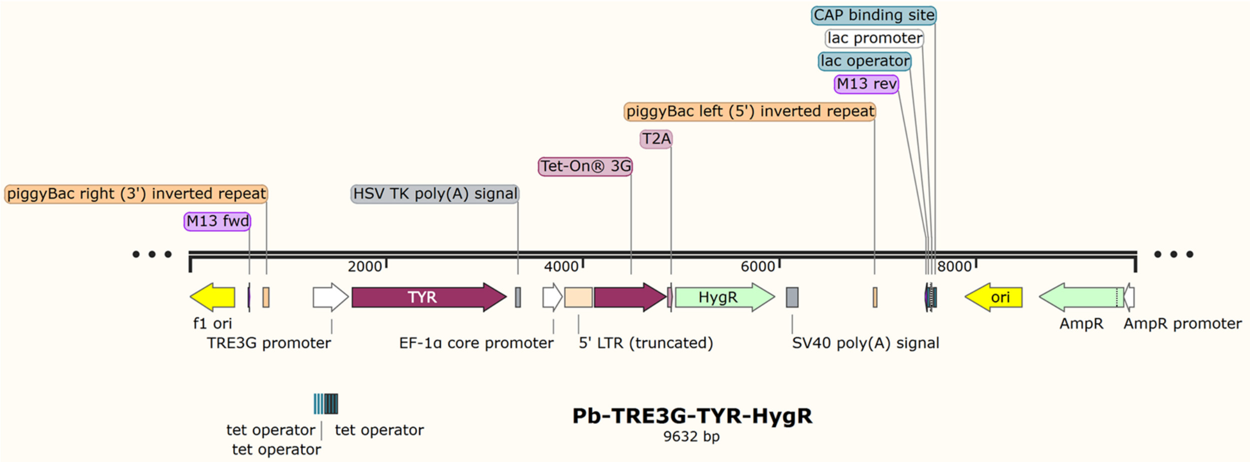

Map of Pb- TRE3G-TYR-HygR plasmid

TYR-G1-G4 knockout cell lines were transfected with the Pb-TRE3G-TYR-HygR plasmid and piggyBac transposase (PB210PA-1, System Biosciences) in a 1:3 ratio using lipofectamine following the manufacturer's instructions. Cells were selected with 40µg/ml hygromycin (InvivoGen, Cat# ant-hg-1) for a week followed by 2µg/ml doxycycline treatment for a month to re-express *TYR* on *TYR* knockout cell lines. Afterwards, cells were lysed for melanin quantification and SSC measurements.

<u>Lentiviral *COMMD3 and RAP2A expression*</u>

Lentiviral RAP2A overexpression plasmid (pCHA1.1-Rap2a-V5) was purchased from Addgene (#149509). Blasticidin cassette was replaced with hygromycin selection cassette using BstXI and KpnI restriction enzymes to generate pCHA1.1-Rap2a-V5-hygro vector (Addgene # 195510). A COMMD3 coding sequence (COMMD3_NM_012071.4) was PCR amplified using forward (ataGCTAGCATGGAGCTCTCGGAGTCTG) and reverse (ataGCGGCCGCTTACAACTGAGTTGCTCTTTCC) primers and cloned into a pCHA1.1-Rap2a-V5-hygro vector using NheI and NotI restriction sites to generate pCHA1.1-COMMD3_CDS-V5-hygro (Addgene# 195509). These two plasmids were packaged into a lentivirus as described earlier (*65*). *COMMD3-KO* and *WT* (control sgRNA edited) clonal lines were generated by plating single cells of bulk *COMMD3-KO* and *WT* (Control-edited) cell populations. Multiple clones were screened for biallelic deletions and confirmed by western blot for COMMD3 using anti-COMMD3 antibody (Bethyl Laboratories, A304-092A).

Endogenous tagging of KLF6 locus with FKBP12^{F36V} and V5

For endogenous tagging of KLF6 at the c-terminus, a dsDNA gene block containing tandem FKBP12^{F36V} and V5 epitope tags was purchased from IDT. The upstream and downstream homologous sequences to the c-terminus of KLF6 were amplified using the following primers: aactccatcactaggggttccggcggccgcCCGGCAGGCTGACACCTCATCCCGCAAGCA, tcctggggagatggtttccacctgcactccGAGGTGCCTCTTCATGTGCAGGGCCAGGTG, CACCTGGCCCTGCACATGAAGAGGCACCTCggagtgcaggtggaaaccatctccccagga, GCCTACAGGATCCACCTCTCTGCTCCCTCAggaaccggatccagagcctgaaccggaggt, acctccggttcaggctctggatccggttccTGAGGGAGCAGAGAGGTGGATCCTGTAGGC, aactccatcactaggggttcctgcggccgcAATTCAAATTCAGAATCATTTAAAAATAAT. These sequences were cloned into an AAV6 donor vector (*67*) (a gift from Matthew Porteus, Stanford University) using Gibson assembly. 293T cells were transfected with plasmids mixed in a 3:1:1 ratio (AD5 helper plasmid: rAAV transgene transfer plasmid: Capsid DJ plasmid). 72 hours post transfection, 293T cells were collected and AAV particles were purified using AAVpro Purification Kit Midi (Takara Bio, Cat# 6675). A chemically modified sgRNA (CACAUGAAGAGGCACCUCUG) targeting the c-terminus of KLF6 was purchased from Synthego (CRISPRevolution sgRNA EZ Kit, Synthego). sgRNA and Cas9 (Integrated DNA Technologies) were mixed per manufacturer's recommendations to assemble a ribonucleoprotein (RNP) complex. MNT-1 cells were nucleofected with KLF6 sgRNA-Cas9 RNP using the Amaxa electroporator (Lonza) with the U-24 program. Nucleofected cells were plated in MNT-1 cell culture medium containing AAV6 donor HDR templates. Single cell colonies were grown, genomic DNA was isolated, the desired region was PCR amplified, and the sequence was verified by Sanger sequencing. Thus, derived homozygous and heterozygous tagged clonal lines were tested for their ability to degrade KLF6 upon dTAG-13 or dTAG^{v}-1. Clonal cells were treated with 500nM dTAG^{v}-1 or 500nM dTAG-13 for 24 hours, as well as DMSO vehicle control and subsequently, protein lysate was collected. Western blot using anti-V5 tag antibody (Abcam, ab15828) was used to confirm KLF6 depletion.

Total cellular melanin quantification

Total melanin quantification was done as described previously (*68*). For primary human melanocytes and PSC-derived melanocytes, $2.5x10^5$ cells were counted, centrifuged at 4000xg for 5 min and washed twice with PBS. Then the cell pellet was dissolved in 250 µl 1M NaOH with 10% dimethyl sulfoxide (DMSO). For the MNT-1 melanoma cell line, $6.0x10^5$ cells were dissolved in 120µl 1M NaOH with 10% dimethyl sulfoxide (DMSO). Cell lysate was incubated at 80ºC for 2 hours. Afterwards, 100 µl of lysate was placed in a 96 well plate to measure absorbance at 400 nm wavelength on an Infinite 200 Pro plate reader (Tecan). A standard curve was generated using synthetic melanin (Sigma) and melanin concentration was determined by reading OD 400 nm against the standard curve (*69*).

Immunocytochemistry and Imaging

Immunocytochemistry was performed as described previously (*66*) . Briefly, cells were fixed in 4% paraformaldehyde, permeabilized with PBS with 0.1% Triton X-100 and blocked with 1%BSA/0.01% Triton X-100. Afterwards, cells were incubated with one of the following anti-

human primary antibodies diluted in blocking buffer: goat PAX3 (1:100; 4ºC overnight; Santa Cruz, sc-34916), mouse TYRP1 (MEL-5) (1:500; 4ºC overnight; BioLegend, 917801), rabbit MITF (1:200; 4ºC overnight; Abcam, ab59232), goat SOX10 (1:50; 4ºC overnight; Santa Cruz, sc-17342), mouse HMB45 (1:20; 4ºC overnight; Life Technologies, 081050), ATP7A (1:100; 4ºC overnight; Santa Cruz, sc-376467). Afterwards, cells were incubated with anti-mouse or anti-goat Alexa Fluor 488 and Alexa Fluor 594 antibodies (1:400; 1 hr at RT; Invitrogen) and counter-stained with DAPI nuclear dye (0.5 µg/ml in PBS; 10 min; Sigma). Cells incubated with secondary antibodies alone served as negative controls. Images were acquired on either an inverted microscope (Nikon Ti2) or on a confocal microscope (Leica TSC SP2). For human and mouse tissue histology, tissues were fixed in 10% neutral buffered formalin and Fontana-Masson and hematoxylin & eosin staining were performed at the Stanford University Pathology Core facility.

LysoTracker Red DND-99 labeling

For melanosomal LysoTracker dye labeling experiments, COMMD3-KO and WT (control-edited) cells were treated for 30 mins with medium containing 50nM LysoTracker Red DND-99 (Life Technologies, Cat# L7528). After 30 mins, cells were washed three times with PBS and fresh medium was replenished. Cells were imaged in a Nikon Spinning Disk Confocal Microscope at 100x/60x magnification. For Lysotracker Red and TYR co-staining experiments, a previously described protocol was followed (*70*). To this end, COMMD3-KO and WT (control-edited) cells were first treated with 50nM LysoTracker Red for 30 min,then fixed with 4% paraformaldehyde, permeabilized with PBS with 0.1% Triton X-100, and blocked with 1% BSA/0.01% Triton X-100. Afterwards, cells were incubated overnight with anti-TYR antibody (MyBioSource, MBS127217) diluted in blocking buffer. Next day, cells were washed and treated with Alexa Fluor 647 antibodies (1:400; 1 hr at RT; Invitrogen), counter-stained with DAPI nuclear dye (0.5 µg/ml in PBS; 10 min; Sigma), and imaged on a Nikon spinning disk confocal microscope at 100x or 60x magnification. Cells incubated with secondary antibodies alone served as negative controls.

Colocalization analysis

Colocalization analysis of ATP7A and TYR co-immunostaining and LysoTracker Red and TYR co-staining were done using the JaCoP plugin in ImageJ (*71*). To this end, three clonal cell lines of COMMD3-KO and WT (control-edited) were stained as described above and images were acquired under identical conditions using a Nikon spinning disk confocal microscope. Each image was split in red (for LysoTracker Red) and green (TYR, Alexa Fluor 647, pseudo-colored as green) channels and converted to 8-bit grayscale images. A threshold of 60 was applied to both channels, and Manders' colocalization coefficients M1 (fraction of red fluorescence in areas with green fluorescence) and M2 (fraction of green fluorescence in areas with red fluorescence) were calculated. For each clone of COMMD3-KO and WT (control-edited), at least four of the analyzed images (total 12-13 images) included 90-100 cells for ATP7A and TYR co-immunostaining experiments and 120-130 cells for TYR and LysoTracker Red co-staining experiments.
M1 and M2 are defined in the following manner by Mander et al., (1993) (*72*): $M1 = (\sum_i R_{i,col}) / \sum_i R_i$ where $R_{i,col} = R_i$ if $G_i > 0$ and $R_{i,col} = 0$ if $G_i = 0$ and $M2 = (\sum_i G_{i,col}) / \sum_i G_i$ where $G_{i,col} =$

Gi if Ri > 0 and $Gi_{,col}$ = 0 if Ri = 0. Where Ri and Gi are the grayscale values of voxel i of the red channel and the green channel. The M2 coefficient, which measured the fraction of TYR positive melanosomes colocalized with ATP7A in the ATP7A and TYR co-immunostaining experiment is shown in Figure S10B. The M2 coefficient, which measured the fraction of TYR positive melanosomes colocalized with LysoTracker Red, indicative of acidic TYR positive melanosomes, is shown in Figure S10F.

Melanosome pH modulation

For melanosomal pH modulation experiments, we used vacuolar-type H+-ATPase (V-ATPase) proton pump inhibitors (bafilomycin A1 and concanamycin) as well as chloroquine, a weak base, which accumulates in endosomal organelles and raises their pH. Clonal COMMD3-KO or *WT* (control-edited) cell lines were treated with either 0.1µm bafilomycin A1 (BafA1),0.1µm concanamycin (ConA), or 50µm chloroquine for 24 hours. Cells were treated with an equivalent amount of DMSO (for BafA1 and ConA) or H2O (for chloroquine) as well, which served as vehicle controls. Cells were then washed twice with PBS and medium was changed with fresh medium containing 50 nM LysoTracker Red dye for 30 min. Afterward, cells were washed three times with PBS and imaged under a Nikon spinning disk confocal microscope or analyzed in a FACSAria flow cytometer (BD Biosciences). In parallel, cells were treated as described above and lysed with 1M NaOH with 10% DMSO for total melanin quantification.

Transmission election microscopy

Cells were cultured on ACLAR coverslips and fixed with 2% glutaraldehyde and 4% paraformaldehyde in 0.1M Sodium Cacodylate buffer, pH 7.4, for 1 hour at room temperature. Thin sections were cut and post-fixed with 1% Uranyl acetate. Sections were subsequently dehydrated, Epon-infiltrated, and examined with a JEOL JEM-1400 series Transmission Electron Microscope at the Stanford Cell Science Imaging Facility. TEM images were acquired for different gene knockouts as well as control-edited cells and melanosomes at various stages of maturation, i.e., stage I-IV were counted in a blinded experiment where the person doing the counting was unaware of the experiment design and expected outcome.

Western blotting

Cas9-MNT-1 cells were induced for 48 hours with 2µg/ml doxycycline to express the Cas9 nuclease. Afterwards, whole cell extracts were obtained by lysing cells in ice cold lysis buffer (300 mM NaCl, 100 mM tris (pH 8), 0.2 mM EDTA, 0.1% NP-40, 10% glycerol, 1 mM phenylmethylsulfonyl fluoride (PMSF), and protease inhibitor cocktail (complete mini, Roche Applied Sciences, Cat# 11836153001) for 20 mins at 4°C. The supernatant was collected, the protein concentrations determined by Bradford assay using Bradford reagent (Bio-Rad), and serial dilutions of proteins were separated on an SDS–polyacrylamide gel electrophoresis (SDS-PAGE) gel. Afterwards, proteins were transferred to nitrocellulose membranes and immunoblotted using mouse monoclonal Cas9 antibody (7A9-3A3, Cell Signaling Technology), mouse monoclonal anti-TYR antibody (Santa Cruz, sc-20035) HRP-conjugated rabbit monoclonal GAPDH antibody (D16H11, Cell Signaling Technology) and HRP-conjugated

mouse monoclonal anti-beta actin antibody (AC-15, Abcam). Protein bands were detected using the Lumi-Light (Roche) chemiluminescence kit as per the manufacturer's instructions.

Melanosome Immunoprecipitation

Melanosome Immunoprecipitation experiments were done as described earlier (32). To this end, MNT-1 cells were transduced with Melano-Tag (GPR143-mScarlet-3xHA, Addgene #160369) or Control-tag (GPR143-mScarlet-1xMyc, Addgene #160368) lentiviral vectors. Melano-Tag localizes on the melanosomal membrane and after gentle cell lysis, intact melanosomes can be immunoprecipitated using anti-HA magnetic beads. Cells transduced with the Control-tag and immunoprecipitated with anti-HA magnetic beads will serve as control. For Melanosome-IP experiments, 10^7 MNT-1 cells were plated per 15-cm plate and collected 3 days after plating. Cell culture medium was changed 2 h before cell harvesting. Cells were washed twice with ice cold PBS,collected by scraping in cold KPBS buffer (10 mM $KH2PO4$, 136 mM KCl, pH 7.25) and pelleted by centrifugation at 2,000g for 2 min at 4 °C. The cell pellet was resuspended in 200 µl cold KPBS buffer and10 µl of the cell suspension was taken aside as input control. Cells were homogenized with a hand-held tissue grinder (VWR) for 30s. 800 µl of KPBS buffer was further added and the suspension was centrifuged at 3,000g for 2 min at 4 °C. Supernatants were then transferred into 1.5-ml tubes that contained 100 µl anti-HA magnetic beads (ThermoFisher Scientific, Cat# 88837) washed with KPBS buffer. The cell suspension was incubated with anti-HA magnetic beads for 10 mins at 4 °C with gentle rocking. Magnetic beads were washed 3 times with 1ml ice-cold KPBS, being transferred to a new collection tube after each wash. Immunoprecipitated and input samples were lysed in 50 µl lysis buffer containing 40 mM HEPES, pH 7.4; 1% Triton-X100; 2.5 mM MgCl2; 10 mM β-glycerol phosphate, 10 mM pyrophosphate, and Thermo Scientific Halt Protease Inhibitor (ThermoFisher Scientific, Cat# 78430). Immunoblotting on input and immunoprecipitated samples was done using antibodies for melanosomal markers PMEL (Santa Cruz, sc-377325) and LAMP2 (Santa Cruz, sc-18822), mitochondrial marker VDAC1 (Cell Signaling Technologies, #4461), as well as COMMD3 (Bethyl Laboratories, A304-092A).

Genome-wide CRISPR screen

MNT-1 cells were transfected using Lipofecatime 2000 with piggyBac transposase (PB210PA-1, System Biosciences) and a piggyBac plasmid expressing SpCas9 under a doxycycline inducible promoter and containing a blasticidin selection cassette. Clonal cells were selected with 5µg/ml blasticidin treatment for a week. These cells, termed Cas-MNT-1 cells, expressed SpCas9 upon doxycycline treatment and were used for the genome-wide CRISPR screen.
The pMCB320 vector-based 10-sgRNA per gene CRISPR-Cas9 library base has been described previously (*20*). This library consists of nine sub-libraries containing ~ 200,000 guides targeting over 20,000 genes and 13,500 control guides. Control guides included non-targeting guides (i.e., no binding sites in the genome) and safe-targeting control guides targeting genomic locations with no annotated functions (*20*).
A large batch of nine CRISPR-Cas9 lentiviral sub-libraries was packaged using methods described previously with some modifications (*65*). Briefly, 293T packaging cells were transfected with CRISPR-Cas9 sub-libraries plasmid and a third-generation lentiviral packaging mix (1:1:1 mix of pMD2.G, pRSV-Rev, and pMDLg/pRRE) in a 1:1 ratio using

polyethylenimine. After 12 hours of transfection, fresh medium (high glucose DMEM plus 10% fetal bovine serum) was supplied to the 293T cells and subsequently two batches of lentivirus were collected at 24 hours and 48 hours. These two batches were combined, passed through a 0.45 um syringe filter (Millipore), ultracentrifuged (50,000xg at 4C for 2 hours) and the viral pellet was resuspended in opti-MEM medium and stored in -80C until further use. Cas9-MNT-1 cells were transduced with serially diluted lentiviral sub-libraries and FACS analyzed for mCherry positive cells to determine transduction efficiency and multiplicity of infection (MOI) calculations. For two independent genome-wide screens, an MOI of 0.1 was used to infect ~200x10^6 Cas9-MNT-1 cells. Cells were selected with 1µg/ml puromycin for two weeks to achieve more than 90% mCherry positive cells and were then treated with 2µg/ml doxycycline for two weeks for Cas9 induction and subsequent gene editing. At this point, cells from the top and bottom 10% of the SSC distribution were FACS sorted on FACSAria cell sorters (BD). For each replicate, ~50x10^6 low SSC and ~50x10^6 high SSC fractions were sorted, totaling ~100x10^6 cells at 500x coverage. Genomic DNA was isolated from 200x10^6 cells using the QIAamp DNA blood midi kit (Qiagen, Cat# 51183) followed by DNA purification using the DNeasy PowerClean Pro Cleanup kit (Qiagen, Cat# 12997-50) to remove coprecipitated melanin following the manufacturer's instructions.

PCR amplification of inserted sgRNAs from the genomic DNA was done with the Herculase II Fusion DNA Polymerase (Agilent Technologies, Cat# 600679) using the primers oMCB-1562 and oMCB-1563. All of the genomic DNA was PCR amplified using the following PCR protocol: 1× 98°C for 2 min, 18× 98°C for 30 sec, 59.1°C for 30 sec, 72°C for 45 sec, 1× 72°C for 3 min. Each PCR reaction included 300 ng of genomic DNA, 20 µL 5X Herculase buffer, 2.5 µL of 10 mM dNTPs, 1 µL 100 µM oMCB-1562, 1 µL 100 µM oMCB-1563, 0.5 µL Herculase II Fusion DNA polymerase, and water to make up the volume to 100 µL. PCR amplicons from each PCR reaction for each group were pooled together and a second five step PCR reaction was set up for each group as follows: 5 µL amplicon from PCR reaction 1, 20 µL 5X Herculase buffer, 2.5 µL of 10 mM dNTPs, 1 µL 100 µM oMCB-1439, 1 µL 100 µM of a unique barcoded primer, 0.5 µL Herculase II Fusion DNA polymerase, and water to make up the volume to 100 µL. PCR conditions for the second PCR reaction were: 1× 98°C for 2 min, 19× 98°C for 30 sec, 59.1°C for 30 sec, 72°C for 45 sec, 1× 72°C for 3 min. PCR amplicons from each group were pooled, and 50 µL was run on a 1% TAE-agarose gel. PCR bands were excised and purified using a Qiagen Gel Purification Kit as per manufacturer's instructions. The sgRNA libraries were sequenced by deep sequencing on an Illumina Hi-Seq using the custom sequencing primer oMCB-1672. Computational analysis of the screen was done using the CasTLE maximum likelihood estimator, which utilizes the background of negative control guides RNAs as a null distribution model against which gene effect sizes, confidence scores, and *P* values are determined. We used safe-targeting guides as control guides in our analysis. The results of the screen are shown in table S1. We performed a search on a pigmentation database (6) in order to classify screen hits as either known or novel pigmentation genes. GO term enrichment analysis of CRISPR screen hits was performed using Gene ontology tools GOrilla (*73*) and REVIGO (*74*). Screen hits at 10% FDR were used as the input gene set in GOrilla for GO term enrichment analysis for biological processes and components. The enriched GO terms with FDR corrected *P* value <0.05 were then used in REVIGO to remove the redundant GO terms and generate xgmml graph files, which were used in Cytoscape software (v3.8.0) to visualize graphs (**Fig. S3**).

Animal Studies

All animals' studies were approved by Stanford University IACUC. Animals were kept in 12 light/12 dark cycle and had free access to food and water. B6.Cg-Tg(Tyr-cre)1Lru/J (RRID:IMSR_JAX:029788) (51) and C57BL/6J (RRID: IMSR_JAX:000664), mice were purchased from Jackson Labs, and Klf6$^{fl/fl}$ (50) mice were a gift from Sandeep Mallipattu, Stony Brook University. For breeding, TyrCre females were kept with Klf6$^{fl/fl}$ male in a cage, which gave TyrCre:: Klf6$^{fl/+}$ progenies in the first generation. TyrCre::Klf6$^{fl/+}$ males and females were crossed again, which resulted in animals with genotypes *Klf6$^{fl/fl}$*, *TyrCre::Klf6$^{fl/fl}$* , *Klf6$^{fl/+}$*, *TyrCre::Klf6$^{fl/+}$*, *Klf6$^{+/+}$*, *TyrCre::Klf6$^{+/+}$* , with expected Mendelian ratios. As the TyrCre transgene was inserted into the X chromosome (51) that gets randomly silenced in females, we used only male animals from this cross to examine the effects of Klf6 deletion. Animals were genotyped by lysing tail tissue in 75 µl buffer (25mM NaOH / 0.2 mM EDTA) at 98ºC for 1 hour. Then 75 µl of 40 mM Tris HCl (pH 5.5) was added to this and samples were centrifuged at 4000rpm for 3 minutes. A 1 µl undiluted aliquot was used for a 25 µl PCR reaction using DreamTaq polymerase (ThermoFisher Scientific, Cat# EP0701) with the following primers for TyrCre detection (CGGTTATTCAACTTGCACCA, AGTGGCCTCTTCCAGAAATG, TGCGACTGTGTCTGATTTCC, TGATAGTCACTCCAGGGGTTG) and the following for *Klf6$^{fl/fl}$* detection (GTCTCTTGACACCTTGACTATCTCTCC, CAAGAAGCCTTCAGAGAACACC).

RNA-seq

Total RNA was isolated from 1x10^{6} primary melanocytes using an RNA isolation kit (Zymo Research, Cat# R2052) as per the manufacturer's instructions. mRNA was isolated using two rounds of Dynal oligo(dT) bead (Invitrogen, Cat# 61002) purification of 10ug total RNA. The mRNA was fragmented with 10X Fragmentation Buffer (Ambion, Cat# AM8740) for 5 minutes and purified afterwards. First strand cDNA synthesis was carried out using Random Hexamer Primers (Invitrogen, Cat# N8080127) and the SuperScript II enzyme (Invitrogen, Cat# 18064014) followed by second strand cDNA synthesis with RNase H (Invitrogen, Cat# EN0201) and DNA Pol I (Invitrogen, Cat# 18010017). The cDNA was purified with a QIAquick column (Qiagen, Cat# 28104). Sequencing libraries were prepared using the Ovation Ultralow System (Nugen/Tecan, Cat# 9149-A01) following manufacturer's instructions. Sequencing adaptors were cleaned by magnetic bead (Agencourt XP, Cat# A63881) size selection. Libraries were QCed by Qubit and Bioanalyzer (Agilent) and multiplexed in six to eight samples per lane for single-end or paired-end sequencing on Illumina HiSeq 4000 or NEXTseq 500 platforms.

Reads were aligned to a hg38 genome model with hisat2 and an ab initio transcript discovery was performed using stringtie. These gene models were reconciled with human gencode 29 gene models using gffcompare. Read coverage over the models was obtained with featureCounts. Expression values as approximate TPMs were calculated at the transcript level without library fragment length correction and subsequently summed for each gene if multiple transcripts per gene were present. Log TPM + 1 expression values for 2216 most variable genes were used to calculate PCA for exploratory analysis. For gene expression (TPM) and melanin (OD 400 nm) correlation analysis, Spearman's rank correlation coefficients were calculated using the cor() function and *P* values were obtained by corr.test() from library('psych') in R. Obtained *P* values were further FDR corrected with qvalue(). For RNA-seq studies on acute depletion of KLF6

upon dTAG^{v}-1 treatment, we used two homozygous and two heterozygous KLF6-FKBP12^{F36V}-V5 tagged clonal cell lines and treated them with either 500nM dTAG^{v}-1 or equivalent DMSO control for 24 hours. These experiments were done twice i.e., two independent replicates and treated as batches for analysis purposes. Afterwards, total RNA was isolated from 14 samples total (eight dTAG^{v}-1 treated samples and six DMSO treated control samples), and RNA-seq libraries were prepared and sequenced. Differential gene expression analysis was done using the statistical package DESeq2 in R. For DESeq2 analysis, we coded dTAG^{v}-1 and DMSO treatment as continuous variables where homozygous samples treated with dTAG^{v}-1 were coded as 0, heterozygous samples treated with dTAG^{v}-1 as 0.9, and DMSO treated samples as 1, based on the overall effect of gene dosage in principal component analysis.

Skin color heritability enrichment analysis

White British UK Biobank GWAS summary statistics were obtained from the Neale lab website (http://www.nealelab.is/uk-biobank/). This GWAS was conducted using self-reported, categorical skin color traits (Very fair, Fair, Light olive, Dark olive, Brown, Black, see https://biobank.ctsu.ox.ac.uk/crystal/field.cgi?id=1717 for details). While the majority of White British individuals belong to the Very fair and Fair categories, there is nevertheless substantial heritable variation among self-reported skin color categories within that population. GWAS was clumped into lead SNPs at various significance thresholds (5e-8, 1e-6, 1e-4) using plink v1.9, with the following parameters: --clump-p1 (*6*) --clump-p2 (*6*) --clump-r2 0.01 --clump-kb 10000. A White British-specific LD reference panel from Sinnott-Armstrong and Naqvi, 2020 (*75*) was used. NCBI-EBI GWAS Catalog results were accessed in May 2020; all associations with the "skin pigmentation" search term were downloaded, and all genes in the "Mapped gene" column were used as the gene set. Since the top 1000 skin color SNPs in African populations were not pruned or clumped for independence, we primarily assigned each SNP to the closest gene. For stratified LD score regression analyses, annotations were constructed using 100kb up- and downstream of either CRISPR activators, repressors, or both. Coefficient Z-scores and heritability enrichments were calculated relative to the set of baselineLD annotations provided by Finucaine et al. (*38*).

Local heritability estimation for genomic regions

HESS (https://doi.org/10.1016/j.ajhg.2016.05.013) was used to estimate local SNP heritability for sets of genomic regions containing pigmentation-associated loci or genes. Neale lab White British UK Biobank GWAS summary statistics were used, together with LD reference panels provided on the HESS website (https://huwenboshi.github.io/hess/) and approximately independent LD blocks in Europeans from Berisza and Pickrell (10.1093/bioinformatics/btv546). HESS was used to estimate the heritability explained by the subset of independent LD blocks that overlapped either a) genome-wide significant skin color GWAS loci as described above, or b) annotated known pigmentation genes from Baxter et. al, (6) extended on 100kb on either side.

Melanocyte eQTL analysis

We obtained eQTL summary statistics for all SNP-gene pairs from a previously published study of primary melanocytes in European-ancestry individuals (40). We first subsetted all SNP-gene pairs for any of the screen hit genes by p-value < 0.01, and from these considered the SNP with the minimum p-value per gene (such that each screen hit was only assigned to one SNP). In cases where the same SNP was assigned to multiple genes, we only considered the gene with the closest TSS to the SNP. We then intersect this list of SNP-gene pairs with the UK Biobank White British skin color GWAS summary statistics, and further only analyzed SNP-gene pairs for which the SNP showed association with skin color (p < 0.01).

Selection analysis

For selection analysis, we obtained population VCF files for the 1000 Genomes Phase 3 Panel (1000G; ftp://ftp.1000genomes.ebi.ac.uk/vol1/ftp/release/20130502/) (*76*). VCF files were filtered to retain biallelic SNPs, with minor allele frequency > 0.01. VCF manipulation and processing were accomplished with VCFtools (*77*). For each melanin-promoting gene identified in our screen, we considered all SNPs within a +/- 100Kb upstream and downstream flanking regions of the gene body in order to consider SNPs within the regulatory domain of said gene. We define these regions as melanin-promoting genic loci. The gene body was defined as the region between the gene's transcription start site (TSS) and transcription end site (TES) provided in GENCODE V34 basic annotation GTF for GRCh37 genome assembly (*78*).
PBS values per SNP were estimated using Weir and Cockerham F_{ST} [defined in (*79*)] and measured between pairwise comparisons of the three continental super-populations. The continental super-populations used in our analysis included Africans, Europeans and East-Asian. F_{ST} were computed using VCFtools. Negative F_{ST} values were treated as zero. F_{ST} values were further log transformed for PBS computation (*41*). For a given three population tree topology, we computed PBS for population A using the equation

$$PBS_A = \frac{1}{2}(E_{A,B} + E_{A,C} - E_{B,C})$$

where E denotes the log-transformed F_{ST} between the indicated pairwise populations of A,B, and C. For the African and European populations, genome-wide PBS statistics per SNP were plotted as Manhattan plots and the top 0.01% and 0.1% quantile of all PBS statistics were superimposed on said plots, in order to characterize SNPs within pro-melanin genic loci with the most extreme PBS values that should have evolved under positive selection.
We compute empirical p-values for each SNP's PBS statistic using the genome-wide distribution of PBS statistics for all SNPs. When comparing PBS p-values with GWAS effect sizes, the set of SNPs within pro-melanin loci was LD-pruned for approximate independence using plink with --indep-pairwise 50 5 0.01 and the 1000G European super-population LD reference panel. The effect of allele that is increasing in frequency in Europeans as compared to Africans and East Asians (using 1000G super-population allele frequencies) was computed using the above-described White British UKBB skin color GWAS.
Some of the schematic diagrams were created using biorender.com.

Fig. S1.

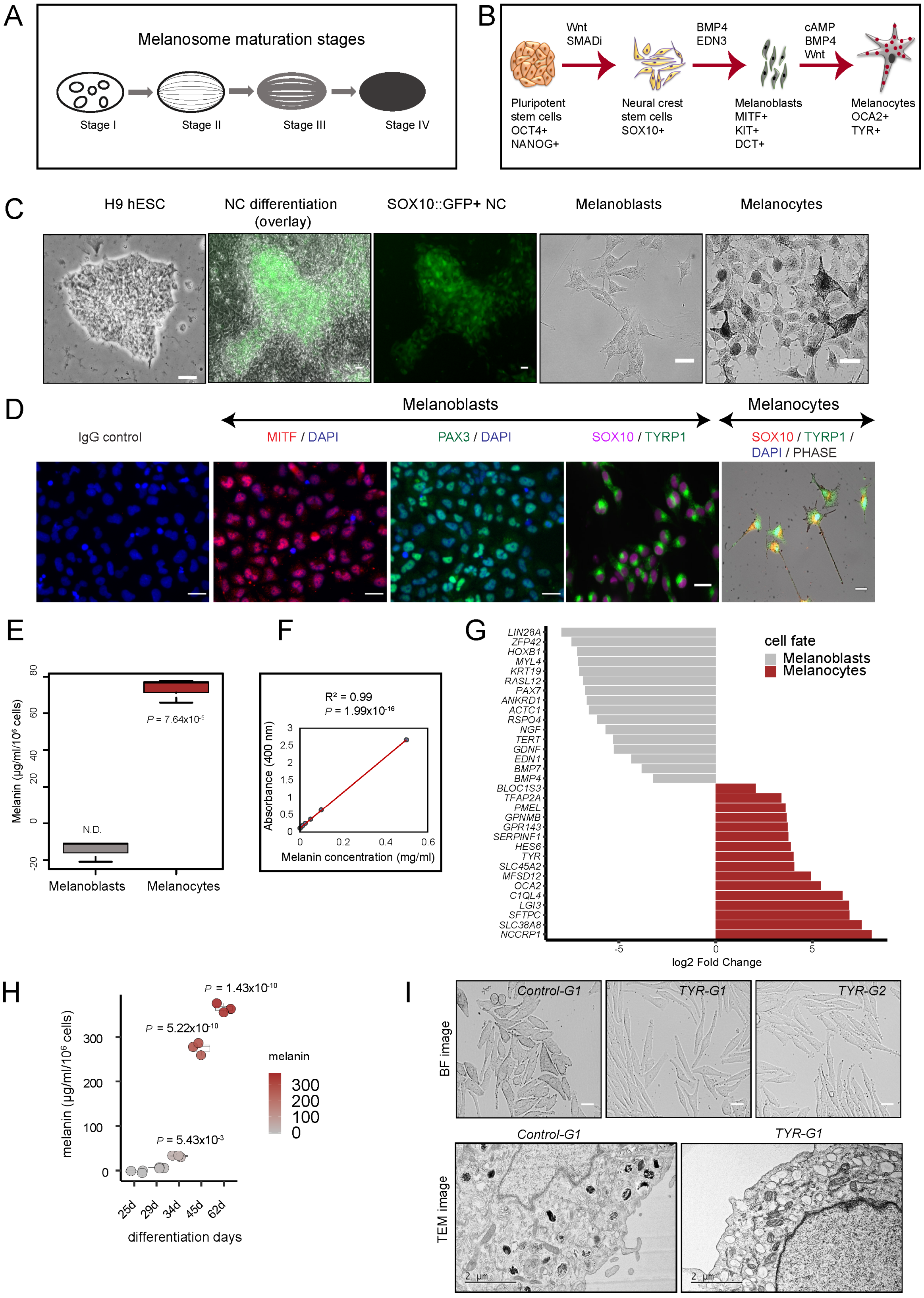
A
Melanosome maturation stages
Stage I
Stage II
Stage III
Stage IV
B
Wnt SMADi
BMP4 EDN3
cAMP BMP4 Wnt
Pluripotent stem cells OCT4+ NANOG+
Neural crest stem cells SOX10+
Melanoblasts MITF+ KIT+ DCT+
Melanocytes OCA2+ TYR+
C
H9 hESC
NC differentiation (overlay)
SOX10::GFP+ NC
Melanoblasts
Melanocytes
D
Melanoblasts
Melanocytes
IgG control
MITF / DAPI
PAX3 / DAPI
SOX10 / TYRP1
SOX10 / TYRP1 / DAPI / PHASE
E
Melanin (μg/ml/10^6 cells)
N.D.
P = 7.64x10^-5
Melanoblasts
Melanocytes
F
R² = 0.99
P = 1.99x10^-16
Absorbance (400 nm)
Melanin concentration (mg/ml)
G
cell fate
Melanoblasts
Melanocytes
LIN28A
ZFP42
HOXB1
MYL4
KRT19
RASL12
PAX7
ANKRD1
ACTC1
RSPO4
NGF
TERT
GDNF
EDN1
BMP7
BMP4
BLOC1S3
TFAP2A
PMEL
GPNMB
GPR143
SERPINF1
HES6
TYR
SLC45A2
MFSD12
OCA2
C1QL4
LGI3
SFTPC
SLC38A8
NCCRP1
log2 Fold Change
H
P = 1.43x10^-10
P = 5.22x10^-10
P = 5.43x10^-3
melanin (μg/ml/10^6 cells)
melanin
25d
29d
34d
45d
62d
differentiation days
I
Control-G1
TYR-G1
TYR-G2
BF image
TEM image
2 μm

555

Fig. S1. Derivation of melanoblasts and melanocytes from pluripotent stem cells (PSC) and melanin measurements. **(A)** Schematic of four stages of melanosome maturation. Stage I melanosomes contain intraluminal vesicles, stage II melanosomes deposit parallel PMEL fibrils on which melanogenesis takes place. Stage III and IV melanosomes correspond to partially and fully melanized states, respectively. (**B)** Schematic of the PSC-to- melanocytes differentiation process. PSC were differentiated into neural crest cells, melanoblasts and melanocytes in a sequential manner. The key growth factors driving differentiation are shown above the arrows, and lineage markers are shown below each cell type. **(C)** Stepwise differentiation of H9 human embryonic stem cells (hESC) *SOX10::GFP* reporter line into neural crest (NC) as shown by GFP+ cells, non-pigmented melanoblasts and finally, pigmented melanocytes. (**D)** Expression of select markers during differentiation is shown: for melanoblast, fate immunostainings for MITF, PAX3, SOX10 and TYRP1; for melanocytes, immunostainings for SOX10 and TYRP1 overlapping with phase contrast image. (**E)** Melanin content of H9 hESC-derived melanoblasts and melanocytes. **(F)** Standard curve depicting relationship between melanin concentration and absorbance at 400 nm. R^2 and *P* value determined by linear regression. (**G)** In comparison to melanoblasts, melanocytes express mature melanogenic genes. The first five genes with the highest log2 fold changes are top 5 most differentially regulated genes in each category. (**H**) Melanin content at different days of differentiation from hESC to melanocytes. Box plots show median and IQR. Significance tested by ANOVA and two-sided pairwise Welch t-Test with BH correction. *P* values shown are relative to day 29 of differentiation. (**I**). Brightfield (BF) and TEM images of *TYR* knockout MNT-1 cells. TEM images show predominance of stage I-II melanosomes and lack of mature melanosomes upon *TYR* loss. (n = 4 sgRNAs treatments). Scale bars: C, D, I (BF) = 25µM. TEM Scale bars: 2 µM. N.D. = not detected.

Fig. S2.

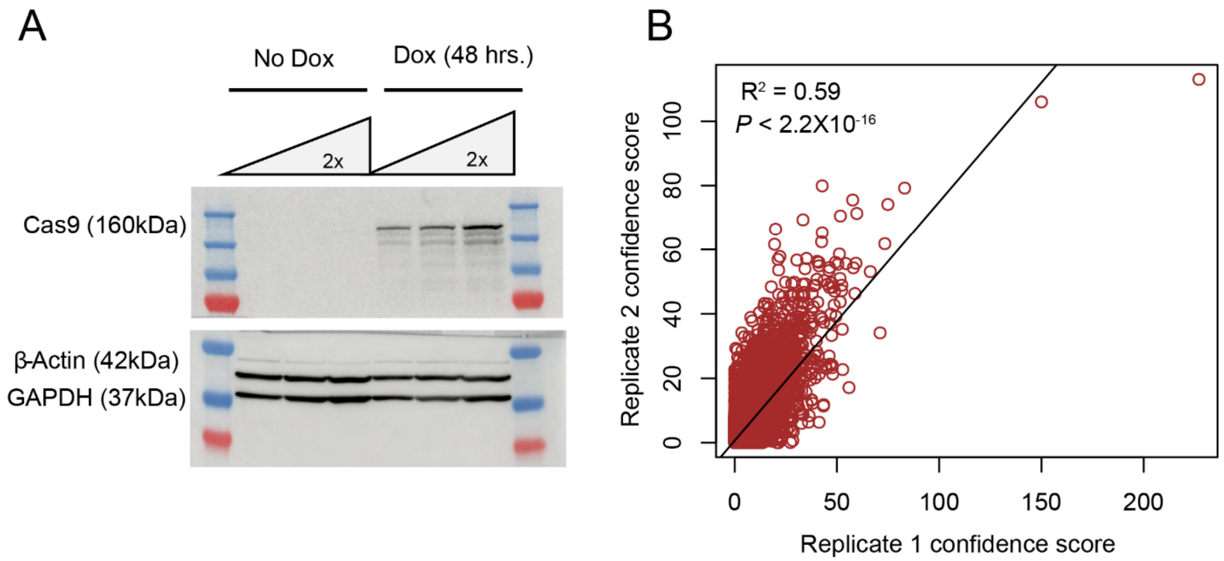

Fig. S2. Inducible Cas9 expressing cell line validation and replication of pigmentation CRISPR-Cas9 screen. (**A**) Western blot for Cas9-MNT-1 cells showing Cas9 expression upon 48-hour doxycycline treatment. (**B**) Reproducibility of two independent genome-wide screens (n=2). CasTLE analysis of genome-wide screens in MNT-1 cells, with CasTLE confidence scores of over 20,000 genes represented as individual points, analyzed by CasTLE likelihood ratio test. R^2 and *P* value determined by linear regression.

Fig. S3.

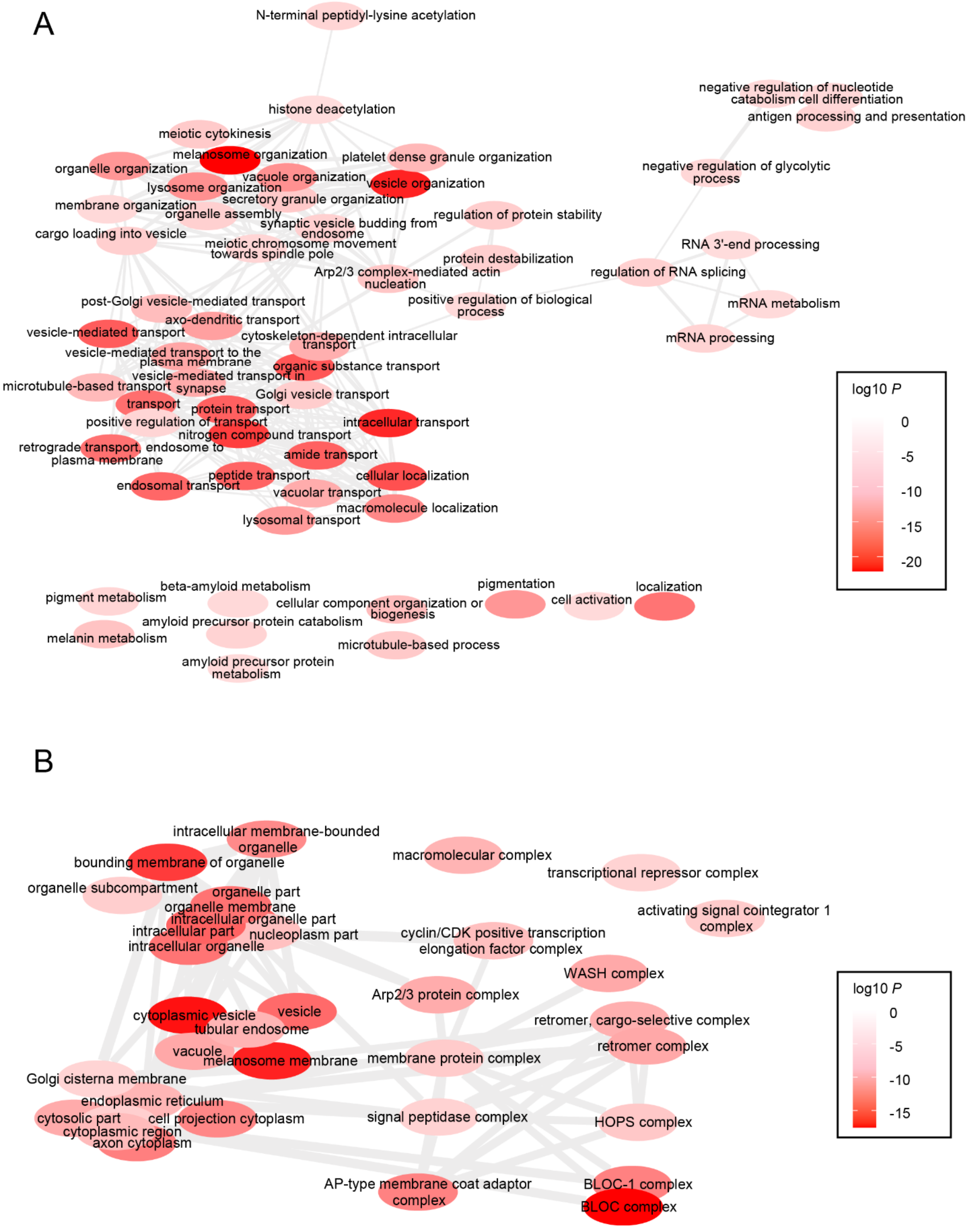
A
N-terminal peptidyl-lysine acetylation
histone deacetylation
meiotic cytokinesis
melanosome organization
platelet dense granule organization
organelle organization
vacuole organization
vesicle organization
lysosome organization
secretory granule organization
membrane organization
organelle assembly
synaptic vesicle budding from endosome
regulation of protein stability
cargo loading into vesicle
meiotic chromosome movement towards spindle pole
protein destabilization
Arp2/3 complex-mediated actin nucleation
positive regulation of biological process
post-Golgi vesicle-mediated transport
axo-dendritic transport
vesicle-mediated transport
cytoskeleton-dependent intracellular transport
vesicle-mediated transport to the plasma membrane
organic substance transport
vesicle-mediated transport in synapse
microtubule-based transport
Golgi vesicle transport
transport
protein transport
positive regulation of transport
intracellular transport
nitrogen compound transport
retrograde transport, endosome to plasma membrane
amide transport
peptide transport
cellular localization
endosomal transport
vacuolar transport
macromolecule localization
lysosomal transport
negative regulation of nucleotide catabolism cell differentiation
antigen processing and presentation
negative regulation of glycolytic process
RNA 3'-end processing
regulation of RNA splicing
mRNA metabolism
mRNA processing
log10 P
0
-5
-10
-15
-20
pigment metabolism
beta-amyloid metabolism
cellular component organization or biogenesis
pigmentation
cell activation
localization
amyloid precursor protein catabolism
melanin metabolism
microtubule-based process
amyloid precursor protein metabolism
B
intracellular membrane-bounded organelle
bounding membrane of organelle
macromolecular complex
transcriptional repressor complex
organelle subcompartment
organelle part
organelle membrane
intracellular organelle part
activating signal cointegrator 1 complex
intracellular part
nucleoplasm part
cyclin/CDK positive transcription elongation factor complex
intracellular organelle
WASH complex
Arp2/3 protein complex
cytoplasmic vesicle
vesicle
tubular endosome
retromer, cargo-selective complex
vacuole
melanosome membrane
membrane protein complex
retromer complex
Golgi cisterna membrane
endoplasmic reticulum
cytosolic part
cell projection cytoplasm
cytoplasmic region
axon cytoplasm
signal peptidase complex
HOPS complex
log10 P
0
-5
-10
-15
AP-type membrane coat adaptor complex
BLOC-1 complex
BLOC complex

Fig. S3. Functional classification of pigmentation screen hits. Gene ontology enrichment analysis for (**A**) biological processes and (**B**) biological components performed using GOrilla (*73*). The screen hits at <10% FDR were used as the input gene set against all genes as background set. Enriched GO terms with q value <0.05 were used as an input in REVIGO (*74*) to remove redundant GO terms and make graphs. Similar GO terms are joined by the edges in the graph, where the line thickness indicates the degree of similarity. Color shading of bubbles represents hypergeometric test *P* values from GOrilla output.

Fig. S4.

A

Known pigmentation genes

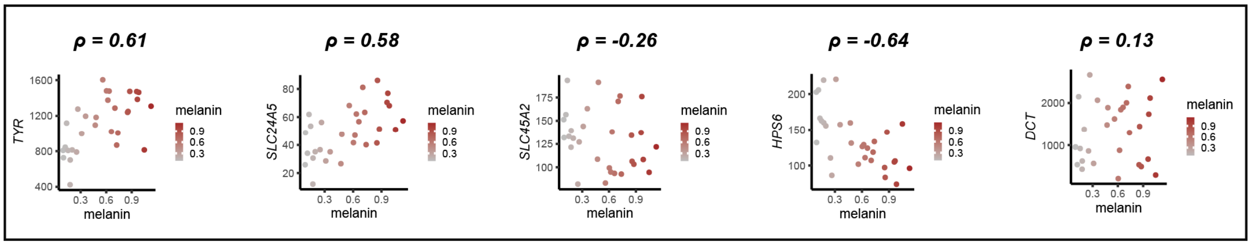

B

Novel pigmentation genes

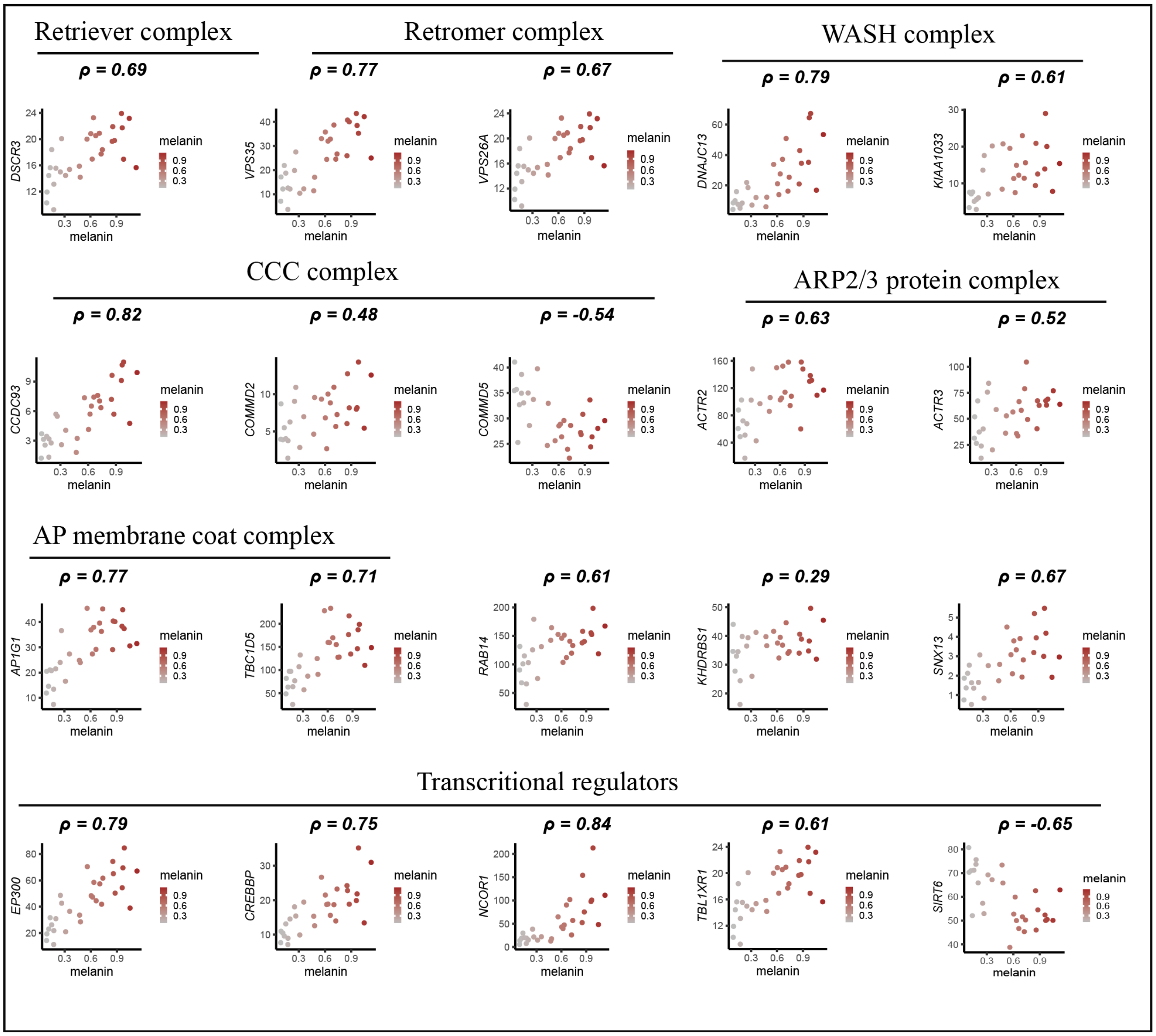

Fig. S4. Spearman's rank correlation coefficients (ρ) depicting relationship between melanin content and gene expression (TPM). (**A)** Select known pigmentation genes rediscovered in the screen. (**B)** Novel melanin-promoting genes classified by their known molecular function. Plotted is melanin content (ordinate) vs RNA-seq expression level (TPM, abscissa).

Fig. S5.

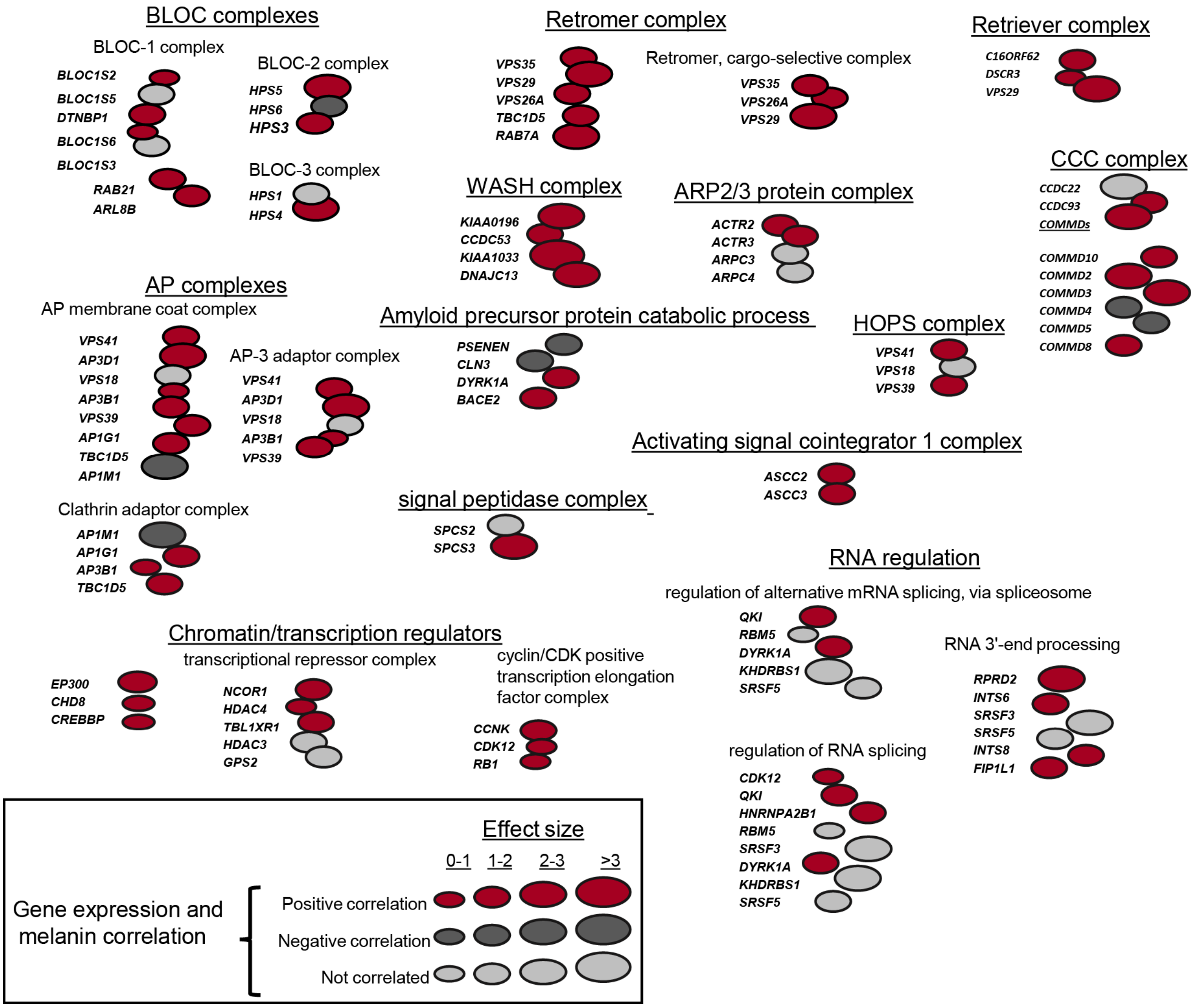

Fig. S5. Classification of screen hits in relation to changes in gene expression in diversely pigmented melanocytes. Schematic of screen hits grouped according to known biological function and presence in common macromolecular complexes (similar to Fig. 2E), further color-coded based on whether there is a positive (dark red), negative (dark grey) or no (light grey) correlation between gene expression (TPM) and melanin content, as detected in the RNA-seq analysis of diversely pigmented individuals. Bubble size indicates CasTLE effect size.

Fig. S6.

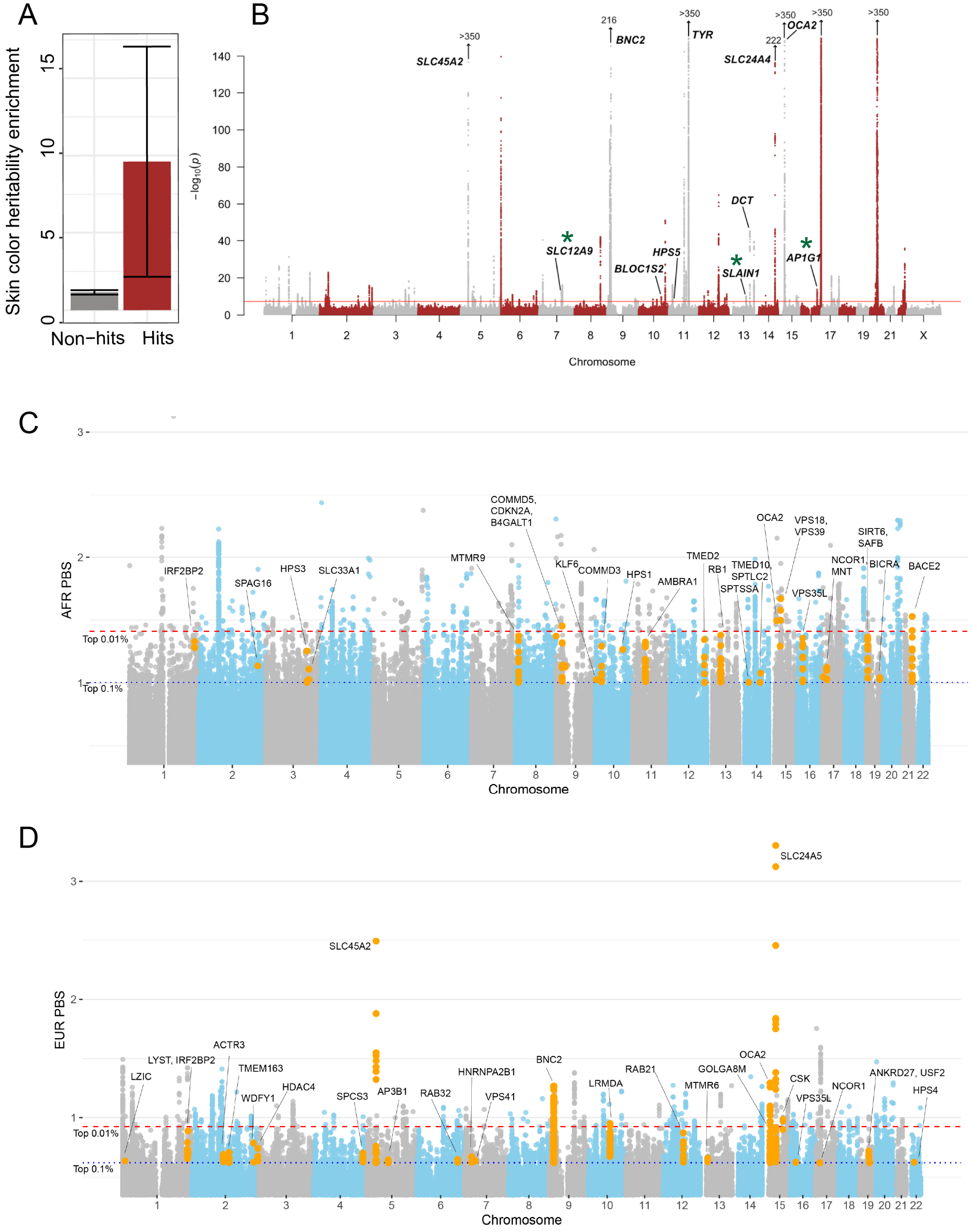
A
B
Skin color heritability enrichment
Non−hits
Hits
SLC45A2
BNC2
TYR
SLC24A4
OCA2
DCT
SLC12A9
HPS5
BLOC1S2
SLAIN1
AP1G1
Chromosome
C
AFR PBS
Top 0.01%
Top 0.1%
IRF2BP2
SPAG16
HPS3
SLC33A1
MTMR9
COMMD5, CDKN2A, B4GALT1
KLF6
COMMD3
HPS1
AMBRA1
TMED2
RB1
TMED10, SPTLC2
SPTSSA
OCA2
VPS18, VPS39
VPS35L
NCOR1, MNT
SIRT6, SAFB
BICRA
BACE2
Chromosome
D
EUR PBS
SLC24A5
SLC45A2
LZIC
LYST, IRF2BP2
ACTR3
TMEM163
WDFY1
HDAC4
SPCS3
AP3B1
RAB32
HNRNPA2B1
VPS41
BNC2
LRMDA
RAB21
MTMR6
GOLGA8M
OCA2
CSK
VPS35L
NCOR1
ANKRD27, USF2
HPS4
Chromosome

640

Fig. S6. Pigmentation screen hits are enriched for skin color heritability and show evidence of recent positive selection. (A) The effect size of skin color heritability enrichment (from UKBB GWAS) for screen hits and non-hits is shown. The screen hits were significantly enriched for skin color heritability (Fold-enrichment 9.61, standard error 3.80, $P = 3.11 \times 10^{-2}$) in comparison to all non-hits (fold-enrichment 1.15, standard error 0.073, $P = 3.37 \times 10^{-2}$). Non-hits are defined as all genes assayed in the CRISPR screen with FDR > 0.1. The Error bars indicate 95% confidence interval for heritability fold-enrichment. (**B**) Manhattan plot of GWAS for skin color in the UK Biobank (UKBB). Genes in bold were detected as pigmentation screen hits and lie within 100kb of a genome-wide significant GWAS signal ($P < 5 \times 10^{-8}$) for skin color. Genes with a green asterisk have no previously described role in pigmentation. Vertical arrows indicate additional SNPs with the indicated -log10(*P*) of association. **(C)** Manhattan plot showing African PBS scores, computed for the African, European, and East-Asian trio. Colored in orange dots are SNPs that reside within 100Kb upstream or downstream of the 169 melanin-promoting genes identified in the screen. Several SNPs within these genic loci have PBS scores that are larger than the top 0.01% quantile of genome-wide PBS scores (i.e., orange dots above the dashed red line), which include *CDKN2A*, *OCA2*, *VPS39*, and *BACE2*. In the more relaxed setting, several melanin-promoting genic loci' SNPs are larger than the top 0.1% quantile of genome-wide PBS scores (orange dots above the blue dotted line). (**D**) Manhattan plot showing European PBS scores, computed for the African, European, and East-Asian trio. *SLC45A2*, *SLC24A5*, *OCA2*, *BNC2*, *GOLGA8M* and *LRMDA* loci have SNPs with PBS scores larger than the top 0.01% quantile of genome-wide threshold (i.e., above the red dashed line). At threshold of 0.1% quantile (blue dotted line), many additional melanin-promoting genes are enriched such as *ACTR3*, *TMEM163*, *HDAC4*, *RAB21*, and *NCOR1*.

Fig. S7.

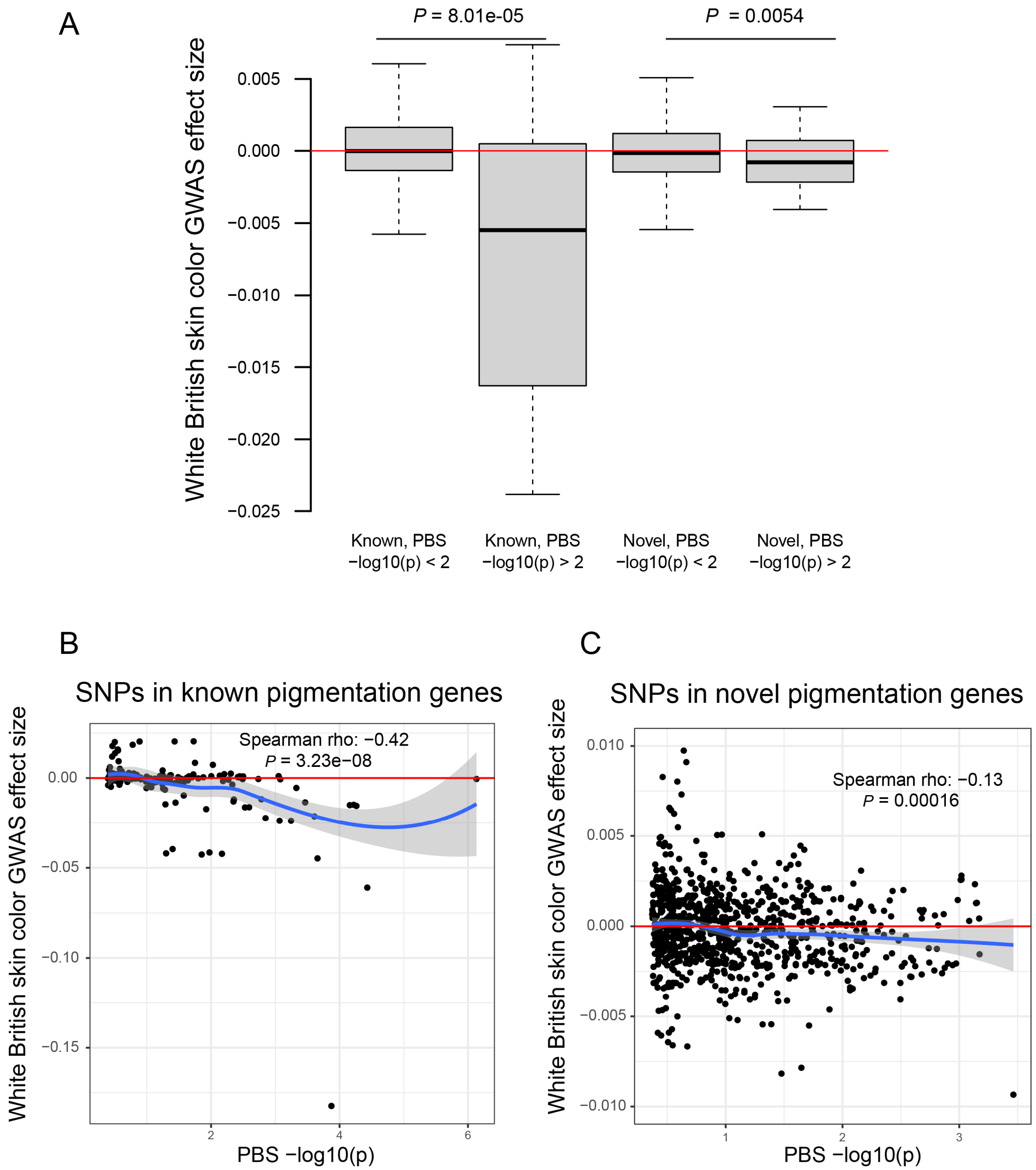
A
P = 8.01e-05
P = 0.0054
White British skin color GWAS effect size
0.005
0.000
−0.005
−0.010
−0.015
−0.020
−0.025
Known, PBS −log10(p) < 2
Known, PBS −log10(p) > 2
Novel, PBS −log10(p) < 2
Novel, PBS −log10(p) > 2
B
SNPs in known pigmentation genes
Spearman rho: −0.42
P = 3.23e−08
White British skin color GWAS effect size
0.00
−0.05
−0.10
−0.15
2
4
6
PBS −log10(p)
C
SNPs in novel pigmentation genes
Spearman rho: −0.13
P = 0.00016
White British skin color GWAS effect size
0.010
0.005
0.000
−0.005
−0.010
1
2
3
PBS −log10(p)

Fig. S7. SNPs of melanin-promoting genes identified in CRISPR screen show evidence of recent positive selection and drive variable melanogenesis in diversely pigmented humans. (**A**) CRISPR screen hits show correlation between selection signals and skin color GWAS effect sizes. For independent SNPs in the indicated PBS -log10(p-value) ranges lying in melanin-promoting gene locus (as defined in Fig. S6C, see Methods) of known (left) or novel (right) screen hits, the effect of the allele that is increased in Europeans on skin color (from UKBB GWAS) is shown. Negative effect sizes mean association with lighter skin color. *P*-values are from Wilcoxon rank-sum test. For independent SNPs lying in the melanin-promoting gene locus of known (**B**) or novel (**C**) screen hits, the effect of the allele that is increased in Europeans on skin color (from UKBB GWAS) is shown. Negative effect sizes mean association with lighter skin color. Blue line and shaded area represent fitted LOESS curve and 95% confidence intervals.

Fig. S8.

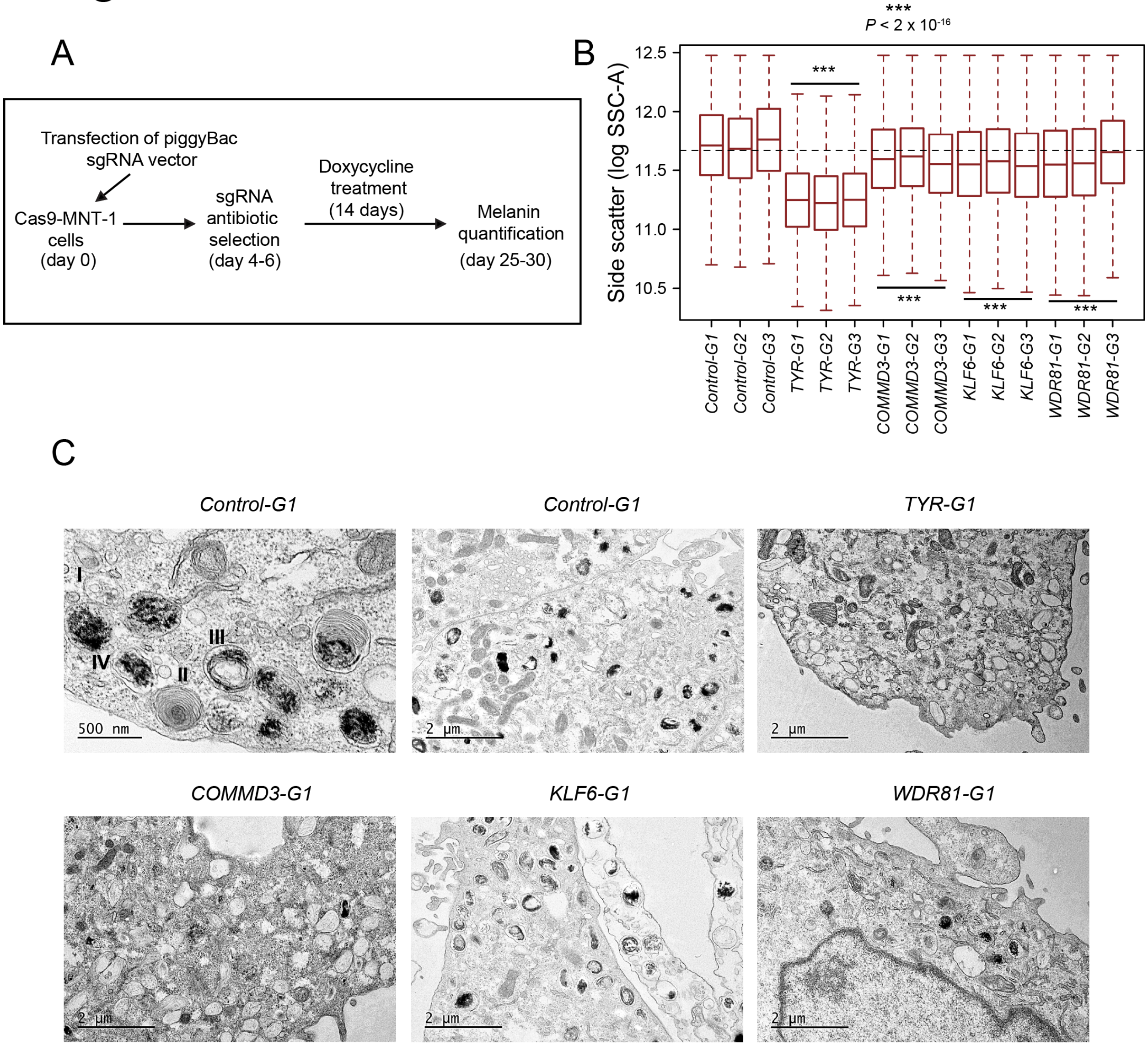

Fig. S8. Effects of candidate gene knockouts on SSC and melanosome biogenesis.

(**A**) Schematic of the CRISPR-Cas9 based validation of novel pigmentation genes. (**B**) Knockouts of candidate genes affect side scatter (SSC) property of cells. Box plots show median log SSC and IQR, whiskers are 1.5 × IQR. Two-sided pairwise Wilcoxon Rank Sum Test with Benjamini & Hochberg correction was performed to compare SSC distributions and significance. *P* values shown are relative to control-edited cells. (**C**) TEM images of indicated gene knockout cells showing distribution of melanosomes. TEM Scale bars: 500 nm and 2 µM.

Fig. S9.

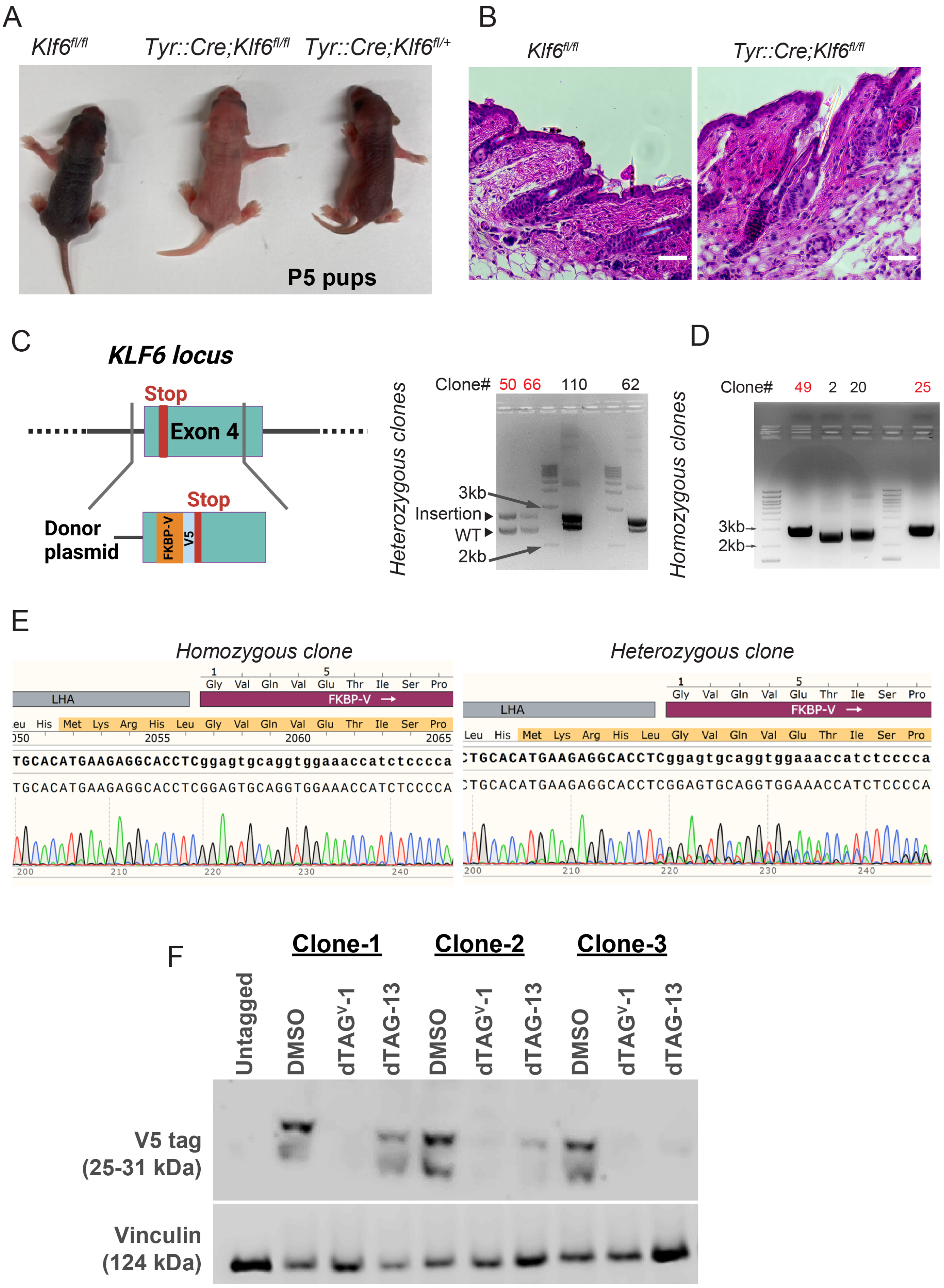
A
Klf6^fl/fl
Tyr::Cre;Klf6^fl/fl
Tyr::Cre;Klf6^fl/+
P5 pups
B
Klf6^fl/fl
Tyr::Cre;Klf6^fl/fl
C
KLF6 locus
Stop
Exon 4
Stop
Donor plasmid
FKBP-V
V5
Heterozygous clones
Clone# 50 66 110 62
3kb
Insertion
WT
2kb
D
Homozygous clones
Clone# 49 2 20 25
3kb
2kb
E
Homozygous clone
Heterozygous clone
LHA
FKBP-V
Gly Val Gln Val Glu Thr Ile Ser Pro
Leu His Met Lys Arg His Leu Gly Val Gln Val Glu Thr Ile Ser Pro
2055 2060 2065
TGCACATGAAGAGGCACCTCggagtgcaggtggaaaccatctcccca
TGCACATGAAGAGGCACCTCGGAGTGCAGGTGGAAACCATCTCCCCA
200 210 220 230 240
F
Clone-1
Clone-2
Clone-3
Untagged
DMSO
dTAG^V-1
dTAG-13
V5 tag (25-31 kDa)
Vinculin (124 kDa)

Fig. S9. KLF6 regulate melanogenesis *in-vivo* and generation of endogenously tagged *KLF6* cell lines. (**A**) P5 pups with both functional *Klf6* alleles (*Klf6*$^{fl/fl}$), no functional *Klf6 alleles* (*TyrCre::Klf6*$^{fl/fl}$), and one functional allele (*TyrCre::Klf6*$^{fl/+}$) show the dose-dependent effect KLF6 has on pigmentation. (**B**) Hematoxylin and eosin-stained mouse skin shows normal skin morphology. (**C**) Schematic diagram of endogenously tagged *KLF6* locus. (**D**) DNA gel electrophoresis images of homozygous (clone #49, #25) and heterozygous tagged clonal cell lines (clone #50, #66) showing correct PCR band sizes. (**E**) Sanger sequencing chromatogram confirming correct sequence of a homozygous and heterozygous tagged clonal line. (**F**) Western blot was performed on cell lysates from three tagged clonal MNT-1 cell lines after 24-hour treatment with DMSO or dTAG molecules (dTAG-13 and dTAG^{v}-1). dTAG^{v}-1 treatment led to rapid degradation of tagged protein as probed by anti-V5 antibody. Scale bars: B, H&E images = 25μM.

Fig. S10.

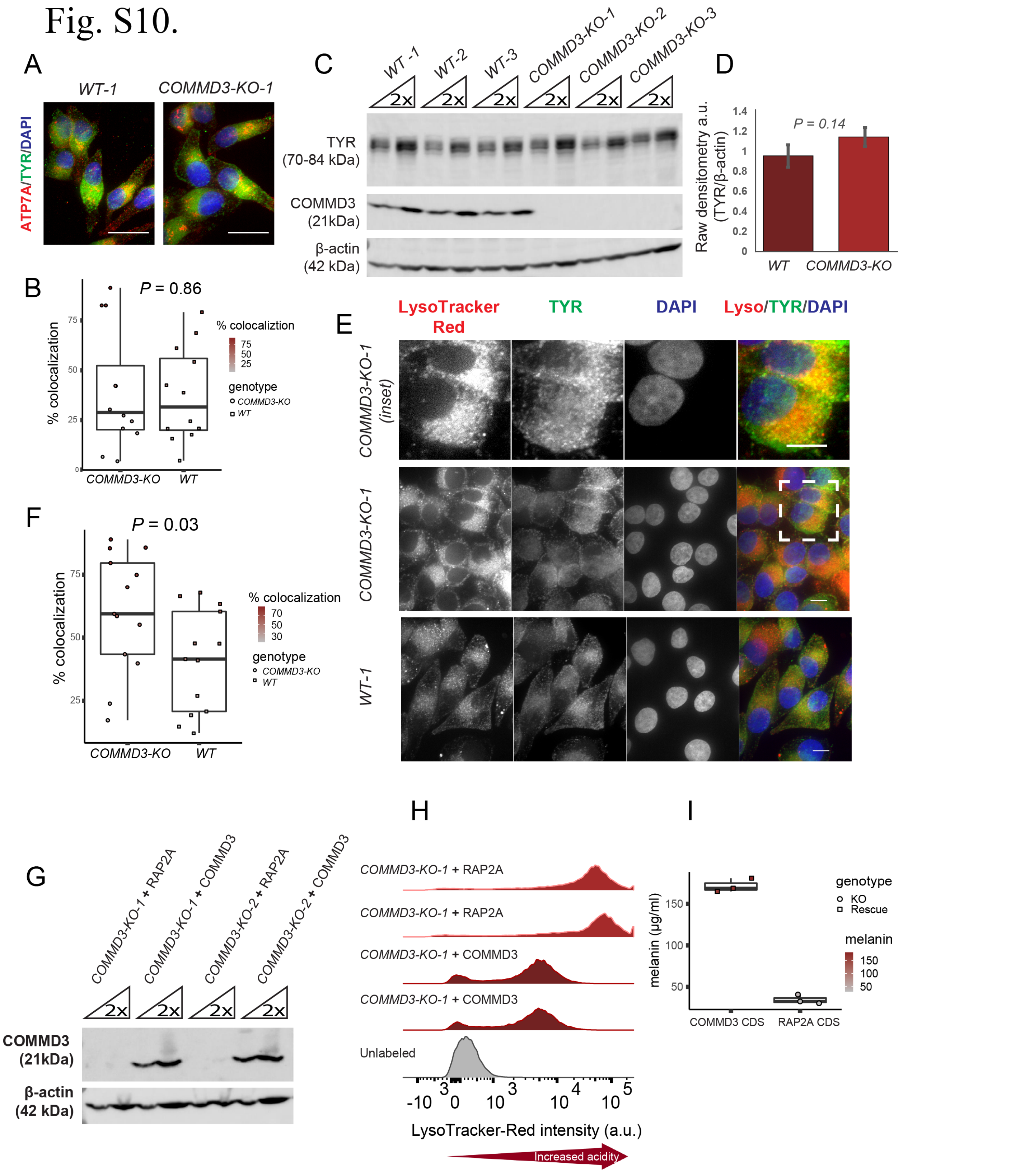
A
WT-1
COMMD3-KO-1
ATP7A/TYR/DAPI
C
WT -1
WT-2
WT-3
COMMD3-KO-1
COMMD3-KO-2
COMMD3-KO-3
2x
TYR
(70-84 kDa)
COMMD3
(21kDa)
β-actin
(42 kDa)
D
Raw densitometry a.u.
(TYR/β-actin)
P = 0.14
WT
COMMD3-KO
B
P = 0.86
% colocalization
% colocaliztion
genotype
COMMD3-KO
WT
E
LysoTracker Red
TYR
DAPI
Lyso/TYR/DAPI
COMMD3-KO-1 (inset)
COMMD3-KO-1
WT-1
F
P = 0.03
% colocalization
genotype
COMMD3-KO
WT
G
COMMD3-KO-1 + RAP2A
COMMD3-KO-1 + COMMD3
COMMD3-KO-2 + RAP2A
COMMD3-KO-2 + COMMD3
2x
COMMD3
(21kDa)
β-actin
(42 kDa)
H
COMMD3-KO-1 + RAP2A
COMMD3-KO-1 + RAP2A
COMMD3-KO-1 + COMMD3
COMMD3-KO-1 + COMMD3
Unlabeled
LysoTracker-Red intensity (a.u.)
Increased acidity
I
melanin (µg/ml)
genotype
KO
Rescue
melanin
COMMD3 CDS
RAP2A CDS

Fig. S10. COMMD3 regulates melanosomal pH and melanogenesis.

(**A**) Colocalization of copper transporter ATP7A in melanosomes is not perturbed by loss of COMMD3, as shown by co-immunostaining of ATP7A and melanosomal marker TYR. (**B**) Colocalization quantification shows no significant difference in ATP7A positive melanosomes in *COMMD3-KO* and *WT* (control-edited) cells. Each circle is Manders' colocalization coefficient M2 from one image, which measures the percentage of TYR fluorescence signal co-occurring with ATP7A fluorescence signal. (**C**) COMMD3 deletion does not affect total TYR protein levels as shown by western blots done on *COMMD3-KO and WT (c*ontrol edited) clonal MNT-1 clonal cell lines (*n=3*). (**D**) ImageJ densitometry quantification of western blots (C) (*n=3*). (**E**) COMMD3 deletion leads to an increase in acidic melanosomes as shown by increased colocalization of TYR positive melanosomes with LysoTracker Red dye (n=3). Images are shown for one *COMMD3-KO-1* and *WT-1* (control-edited) clonal line. (**F**) Colocalization quantification shows a significantly higher percentage of Lysotracker Red-containing (acidic) and TYR positive melanosomes in *COMMD3-KO* cells compared to *WT* (control-edited) cells. Each circle is Manders' colocalization coefficient M2 from one image, which measures the percentage of TYR fluorescence signal co-occurring with LysoTracker Red fluorescence signal. (**G**) Immunoblots for two *COMMD3-KO* clonal lines expressing COMMD3 show a band for COMMD3, while no COMMD3 band is seen when an unrelated protein, RAP2A (*32*), is overexpressed. (**H**) COMMD3 overexpression in *COMMD3-KO* cells rescues reduced pH compared to RAP2A (a constitutively expressed protein) overexpression as shown by flow cytometry histograms. *n=2.* (**I**) COMMD3 overexpression in *COMMD3-KO* cells rescues the loss of melanin as shown by melanin quantification. Box plots show median log absorbance and IQR. Significance tested by Welch t-test. *n=3.* Scale bars: A= 25µM; E= 10 µM.

Table S1. CRISPR screen hits corresponding to low side scatter FACS sort are shown with hits at 10% FDR are highlighted in yellow color and hits between 10-20% FDR are highlighted with blue color.
< Excel file>

Table S2. GO biological processes and components enrichment analysis for CRISPR screen hits at 10% FDR.
< Excel file>

Table S3. Donor records for human foreskin tissue collection. Parent-reported ethnicity and melanin measurements are documented.
< Excel file>

Table S4. RNA-seq transcript per million (TPM) data for all primary human melanocytes. All columns contain donor identifier as described in table S3.
< Excel file>

Table S5. Spearman correlation coefficients calculation for RNA-seq TPM and melanin (OD 400 nm) measurements.
< Excel file>

Table S6. Fisher test contingency table and gene assignment table for human pigmentation heritability enrichment analyses.
< Excel file>

Table S7. Table of African PBS scores and their empirical p-values. Genomic coordinates are hg19. SNP details (i.e., rsid, reference and alternate allele information) were obtained from the 1000G VCFs.
< Excel file>

Table S8. Table of European PBS scores and their empirical p-values. Genomic coordinates are hg19. SNP details (i.e., rsid, reference and alternate allele information) were obtained from the 1000G VCFs.
< Excel file>

Table S9. Animal husbandry genotype and phenotype record.
< Excel file>

Table S10. Skin melanocytes count data for *Klf6*$^{+/+}$ and *TyrCre::Klf6*$^{fl/fl}$ genotyped animals.
< Excel file>

References and Notes

1. W. J. Pavan, R. A. Sturm, The Genetics of Human Skin and Hair Pigmentation. *Annu. Rev. Genomics Hum. Genet.* **20**, 41–72 (2019). doi:10.1146/annurev-genom-083118-015230 Medline

2. E. E. Quillen, H. L. Norton, E. J. Parra, F. Lona-Durazo, K. C. Ang, F. M. Illiescu, L. N. Pearson, M. D. Shriver, T. Lasisi, O. Gokcumen, I. Starr, Y.-L. Lin, A. R. Martin, N. G. Jablonski, Shades of complexity: New perspectives on the evolution and genetic architecture of human skin. *Am. J. Phys. Anthropol.* **168** (suppl. 67), 4–26 (2019). doi:10.1002/ajpa.23737 Medline

3. J. Rocha, The Evolutionary History of Human Skin Pigmentation. *J. Mol. Evol.* **88**, 77–87 (2020). doi:10.1007/s00239-019-09902-7 Medline

4. G. Raposo, M. S. Marks, Melanosomes—Dark organelles enlighten endosomal membrane transport. *Nat. Rev. Mol. Cell Biol.* **8**, 786–797 (2007). doi:10.1038/nrm2258 Medline

5. L. Le, J. Sirés-Campos, G. Raposo, C. Delevoye, M. S. Marks, Melanosome Biogenesis in the Pigmentation of Mammalian Skin. *Integr. Comp. Biol.* **61**, 1517–1545 (2021). doi:10.1093/icb/icab078 Medline

6. L. L. Baxter, D. E. Watkins-Chow, W. J. Pavan, S. K. Loftus, A curated gene list for expanding the horizons of pigmentation biology. *Pigment Cell Melanoma Res.* **32**, 348–358 (2019). doi:10.1111/pcmr.12743 Medline

7. A. R. Martin, M. Lin, J. M. Granka, J. W. Myrick, X. Liu, A. Sockell, E. G. Atkinson, C. J. Werely, M. Möller, M. S. Sandhu, D. M. Kingsley, E. G. Hoal, X. Liu, M. J. Daly, M. W. Feldman, C. R. Gignoux, C. D. Bustamante, B. M. Henn, An Unexpectedly Complex Architecture for Skin Pigmentation in Africans. *Cell* **171**, 1340–1353.e14 (2017). doi:10.1016/j.cell.2017.11.015 Medline

8. N. G. Crawford, D. E. Kelly, M. E. B. Hansen, M. H. Beltrame, S. Fan, S. L. Bowman, E. Jewett, A. Ranciaro, S. Thompson, Y. Lo, S. P. Pfeifer, J. D. Jensen, M. C. Campbell, W. Beggs, F. Hormozdiari, S. W. Mpoloka, G. G. Mokone, T. Nyambo, D. W. Meskel, G. Belay, J. Haut, H. Rothschild, L. Zon, Y. Zhou, M. A. Kovacs, M. Xu, T. Zhang, K. Bishop, J. Sinclair, C. Rivas, E. Elliot, J. Choi, S. A. Li, B. Hicks, S. Burgess, C. Abnet, D. E. Watkins-Chow, E. Oceana, Y. S. Song, E. Eskin, K. M. Brown, M. S. Marks, S. K. Loftus, W. J. Pavan, M. Yeager, S. Chanock, S. A. Tishkoff; NISC Comparative Sequencing Program, Loci associated with skin pigmentation identified in African populations. *Science* **358**, eaan8433 (2017). doi:10.1126/science.aan8433 Medline

9. S. Beleza, N. A. Johnson, S. I. Candille, D. M. Absher, M. A. Coram, J. Lopes, J. Campos, I. I. Araújo, T. M. Anderson, B. J. Vilhjálmsson, M. Nordborg, A. Correia E Silva, M. D. Shriver, J. Rocha, G. S. Barsh, H. Tang, Genetic architecture of skin and eye color in an African-European admixed population. *PLOS Genet.* **9**, e1003372 (2013). doi:10.1371/journal.pgen.1003372 Medline

10. E. Pairo-Castineira *et al.*, Expanded Analysis of Pigmentation Genetics in UK Biobank. bioRxiv 2022.2001.2030.478418 [Preprint] (2022). https://doi.org/10.1101/2022.01.30.478418

11. H. Shi, G. Kichaev, B. Pasaniuc, Contrasting the Genetic Architecture of 30 Complex Traits from Summary Association Data, Contrasting the Genetic Architecture of 30 Complex Traits from Summary Association Data. *Am. J. Hum. Genet.* **99**, 139–153 (2016). doi:10.1016/j.ajhg.2016.05.013 Medline

12. P. Meredith, T. Sarna, The physical and chemical properties of eumelanin. *Pigment Cell Res.* **19**, 572–594 (2006). doi:10.1111/j.1600-0749.2006.00345.x Medline

13. G. C. Salzman, J. M. Crowell, J. C. Martin, T. T. Trujillo, A. Romero, P. F. Mullaney, P. M. LaBauve, Cell classification by laser light scattering: Identification and separation of unstained leukocytes. *Acta Cytol.* **19**, 374–377 (1975). Medline

14. Y. Mica, G. Lee, S. M. Chambers, M. J. Tomishima, L. Studer, Modeling neural crest induction, melanocyte specification, and disease-related pigmentation defects in hESCs and patient-specific iPSCs. *Cell Rep.* **3**, 1140–1152 (2013). doi:10.1016/j.celrep.2013.03.025 Medline

15. K. Hoek, D. L. Rimm, K. R. Williams, H. Zhao, S. Ariyan, A. Lin, H. M. Kluger, A. J. Berger, E. Cheng, E. S. Trombetta, T. Wu, M. Niinobe, K. Yoshikawa, G. E. Hannigan, R. Halaban, Expression profiling reveals novel pathways in the transformation of melanocytes to melanomas. *Cancer Res.* **64**, 5270–5282 (2004). doi:10.1158/0008-5472.CAN-04-0731 Medline

16. A. Chi, J. C. Valencia, Z.-Z. Hu, H. Watabe, H. Yamaguchi, N. J. Mangini, H. Huang, V. A. Canfield, K. C. Cheng, F. Yang, R. Abe, S. Yamagishi, J. Shabanowitz, V. J. Hearing, C. Wu, E. Appella, D. F. Hunt, Proteomic and bioinformatic characterization of the biogenesis and function of melanosomes. *J. Proteome Res.* **5**, 3135–3144 (2006). doi:10.1021/pr060363j Medline

17. I. Hurbain, W. J. C. Geerts, T. Boudier, S. Marco, A. J. Verkleij, M. S. Marks, G. Raposo, Electron tomography of early melanosomes: Implications for melanogenesis and the generation of fibrillar amyloid sheets. *Proc. Natl. Acad. Sci. U.S.A.* **105**, 19726–19731 (2008). doi:10.1073/pnas.0803488105 Medline

18. T. Kushimoto, V. Basrur, J. Valencia, J. Matsunaga, W. D. Vieira, V. J. Ferrans, J. Muller, E. Appella, V. J. Hearing, A model for melanosome biogenesis based on the purification and analysis of early melanosomes. *Proc. Natl. Acad. Sci. U.S.A.* **98**, 10698–10703 (2001). doi:10.1073/pnas.191184798 Medline

19. A. K. Ganesan, H. Ho, B. Bodemann, S. Petersen, J. Aruri, S. Koshy, Z. Richardson, L. Q. Le, T. Krasieva, M. G. Roth, P. Farmer, M. A. White, Genome-wide siRNA-based functional genomics of pigmentation identifies novel genes and pathways that impact melanogenesis in human cells. *PLOS Genet.* **4**, e1000298 (2008). doi:10.1371/journal.pgen.1000298 Medline

20. D. W. Morgens, M. Wainberg, E. A. Boyle, O. Ursu, C. L. Araya, C. K. Tsui, M. S. Haney, G. T. Hess, K. Han, E. E. Jeng, A. Li, M. P. Snyder, W. J. Greenleaf, A. Kundaje, M. C. Bassik, Genome-scale measurement of off-target activity using Cas9 toxicity in high-throughput screens. *Nat. Commun.* **8**, 15178 (2017). doi:10.1038/ncomms15178 Medline

21. D. W. Morgens, R. M. Deans, A. Li, M. C. Bassik, Systematic comparison of CRISPR/Cas9 and RNAi screens for essential genes. *Nat. Biotechnol.* **34**, 634–636 (2016). doi:10.1038/nbt.3567 Medline

22. H. M. Shapiro, *Practical Flow Cytometry* (Wiley-Liss, 2003).

23. T. Hart, M. Chandrashekhar, M. Aregger, Z. Steinhart, K. R. Brown, G. MacLeod, M. Mis, M. Zimmermann, A. Fradet-Turcotte, S. Sun, P. Mero, P. Dirks, S. Sidhu, F. P. Roth, O. S. Rissland, D. Durocher, S. Angers, J. Moffat, High-Resolution CRISPR Screens Reveal Fitness Genes and Genotype-Specific Cancer Liabilities. *Cell* **163**, 1515–1526 (2015). doi:10.1016/j.cell.2015.11.015 Medline

24. R. J. Ihry, M. R. Salick, D. J. Ho, M. Sondey, S. Kommineni, S. Paula, J. Raymond, B. Henry, E. Frias, Q. Wang, K. A. Worringer, C. Ye, C. Russ, J. S. Reece-Hoyes, R. C. Altshuler, R. Randhawa, Z. Yang, G. McAllister, G. R. Hoffman, R. Dolmetsch, A. Kaykas, Genome-Scale CRISPR Screens Identify Human Pluripotency-Specific Genes. *Cell Rep.* **27**, 616–630.e6 (2019). doi:10.1016/j.celrep.2019.03.043 Medline

25. K. Tzelepis, H. Koike-Yusa, E. De Braekeleer, Y. Li, E. Metzakopian, O. M. Dovey, A. Mupo, V. Grinkevich, M. Li, M. Mazan, M. Gozdecka, S. Ohnishi, J. Cooper, M. Patel, T. McKerrell, B. Chen, A. F. Domingues, P. Gallipoli, S. Teichmann, H. Ponstingl, U. McDermott, J. Saez-Rodriguez, B. J. P. Huntly, F. Iorio, C. Pina, G. S. Vassiliou, K. Yusa, A CRISPR Dropout Screen Identifies Genetic Vulnerabilities and Therapeutic Targets in Acute Myeloid Leukemia. *Cell Rep.* **17**, 1193–1205 (2016). doi:10.1016/j.celrep.2016.09.079 Medline

26. G. G. McGill, M. Horstmann, H. R. Widlund, J. Du, G. Motyckova, E. K. Nishimura, Y.-L. Lin, S. Ramaswamy, W. Avery, H.-F. Ding, S. A. Jordan, I. J. Jackson, S. J. Korsmeyer, T. R. Golub, D. E. Fisher, Bcl2 regulation by the melanocyte master regulator Mitf modulates lineage survival and melanoma cell viability. *Cell* **109**, 707–718 (2002). doi:10.1016/S0092-8674(02)00762-6 Medline

27. M. L. Harris, K. Buac, O. Shakhova, R. M. Hakami, M. Wegner, L. Sommer, W. J. Pavan, A dual role for SOX10 in the maintenance of the postnatal melanocyte lineage and the differentiation of melanocyte stem cell progenitors. *PLOS Genet.* **9**, e1003644 (2013). doi:10.1371/journal.pgen.1003644 Medline

28. L. Hou, H. Arnheiter, W. J. Pavan, Interspecies difference in the regulation of melanocyte development by SOX10 and MITF. *Proc. Natl. Acad. Sci. U.S.A.* **103**, 9081–9085 (2006). doi:10.1073/pnas.0603114103 Medline

29. G. Yang, Y. Li, E. K. Nishimura, H. Xin, A. Zhou, Y. Guo, L. Dong, M. F. Denning, B. J. Nickoloff, R. Cui, Inhibition of PAX3 by TGF-beta modulates melanocyte viability. *Mol. Cell* **32**, 554–563 (2008). doi:10.1016/j.molcel.2008.11.002 Medline

30. P. Sulem, D. F. Gudbjartsson, S. N. Stacey, A. Helgason, T. Rafnar, M. Jakobsdottir, S. Steinberg, S. A. Gudjonsson, A. Palsson, G. Thorleifsson, S. Pálsson, B. Sigurgeirsson, K. Thorisdottir, R. Ragnarsson, K. R. Benediktsdottir, K. K. Aben, S. H. Vermeulen, A. M. Goldstein, M. A. Tucker, L. A. Kiemeney, J. H. Olafsson, J. Gulcher, A. Kong, U. Thorsteinsdottir, K. Stefansson, Two newly identified genetic determinants of pigmentation in Europeans. *Nat. Genet.* **40**, 835–837 (2008). doi:10.1038/ng.160 Medline

31. A. L. Ambrosio, J. A. Boyle, A. E. Aradi, K. A. Christian, S. M. Di Pietro, TPC2 controls pigmentation by regulating melanosome pH and size. *Proc. Natl. Acad. Sci. U.S.A.* **113**, 5622–5627 (2016). doi:10.1073/pnas.1600108113 Medline

32. C. H. Adelmann, A. K. Traunbauer, B. Chen, K. J. Condon, S. H. Chan, T. Kunchok, C. A. Lewis, D. M. Sabatini, MFSD12 mediates the import of cysteine into melanosomes and lysosomes. *Nature* **588**, 699–704 (2020). doi:10.1038/s41586-020-2937-x Medline

33. C. A. Phillips-Krawczak, A. Singla, P. Starokadomskyy, Z. Deng, D. G. Osborne, H. Li, C. J. Dick, T. S. Gomez, M. Koenecke, J.-S. Zhang, H. Dai, L. F. Sifuentes-Dominguez, L. N. Geng, S. H. Kaufmann, M. Y. Hein, M. Wallis, J. McGaughran, J. Gecz, Bv. Sluis, D. D. Billadeau, E. Burstein, COMMD1 is linked to the WASH complex and regulates endosomal trafficking of the copper transporter ATP7A. *Mol. Biol. Cell* **26**, 91–103 (2015). doi:10.1091/mbc.e14-06-1073 Medline

34. P. J. Cullen, F. Steinberg, To degrade or not to degrade: Mechanisms and significance of endocytic recycling. *Nat. Rev. Mol. Cell Biol.* **19**, 679–696 (2018). doi:10.1038/s41580-018-0053-7 Medline

35. L. Ripoll, X. Heiligenstein, G. Raposo, C. Delevoye, Illuminating the dark side of recycling endosomes. *Cell Cycle* **15**, 1309–1310 (2016). doi:10.1080/15384101.2016.1160682 Medline

36. H. K. Long, S. L. Prescott, J. Wysocka, Ever-Changing Landscapes: Transcriptional Enhancers in Development and Evolution. *Cell* **167**, 1170–1187 (2016). doi:10.1016/j.cell.2016.09.018 Medline

37. P. J. Wittkopp, G. Kalay, Cis-regulatory elements: Molecular mechanisms and evolutionary processes underlying divergence. *Nat. Rev. Genet.* **13**, 59–69 (2011). doi:10.1038/nrg3095 Medline

38. H. K. Finucane, B. Bulik-Sullivan, A. Gusev, G. Trynka, Y. Reshef, P.-R. Loh, V. Anttila, H. Xu, C. Zang, K. Farh, S. Ripke, F. R. Day, S. Purcell, E. Stahl, S. Lindstrom, J. R. B. Perry, Y. Okada, S. Raychaudhuri, M. J. Daly, N. Patterson, B. M. Neale, A. L. Price; ReproGen Consortium; Schizophrenia Working Group of the Psychiatric Genomics Consortium; RACI Consortium, Partitioning heritability by functional annotation using genome-wide association summary statistics. *Nat. Genet.* **47**, 1228–1235 (2015). doi:10.1038/ng.3404 Medline

39. J. MacArthur, E. Bowler, M. Cerezo, L. Gil, P. Hall, E. Hastings, H. Junkins, A. McMahon, A. Milano, J. Morales, Z. M. Pendlington, D. Welter, T. Burdett, L. Hindorff, P. Flicek, F. Cunningham, H. Parkinson, The new NHGRI-EBI Catalog of published genome-wide association studies (GWAS Catalog). *Nucleic Acids Res.* **45**, D896–D901 (2017). doi:10.1093/nar/gkw1133 Medline

40. T. Zhang, J. Choi, M. A. Kovacs, J. Shi, M. Xu, A. M. Goldstein, A. J. Trower, D. T. Bishop, M. M. Iles, D. L. Duffy, S. MacGregor, L. T. Amundadottir, M. H. Law, S. K. Loftus, W. J. Pavan, K. M. Brown; NISC Comparative Sequencing Program; Melanoma Meta-Analysis Consortium, Cell-type-specific eQTL of primary melanocytes facilitates identification of melanoma susceptibility genes. *Genome Res.* **28**, 1621–1635 (2018). doi:10.1101/gr.233304.117 Medline

41. X. Yi, Y. Liang, E. Huerta-Sanchez, X. Jin, Z. X. P. Cuo, J. E. Pool, X. Xu, H. Jiang, N. Vinckenbosch, T. S. Korneliussen, H. Zheng, T. Liu, W. He, K. Li, R. Luo, X. Nie, H. Wu, M. Zhao, H. Cao, J. Zou, Y. Shan, S. Li, Q. Yang, P. Asan, P. Ni, G. Tian, J. Xu, X. Liu, T. Jiang, R. Wu, G. Zhou, M. Tang, J. Qin, T. Wang, S. Feng, G. Li, J. Huasang, J. Luosang, W. Wang, F. Chen, Y. Wang, X. Zheng, Z. Li, Z. Bianba, G. Yang, X. Wang, S. Tang, G. Gao, Y. Chen, Z. Luo, L. Gusang, Z. Cao, Q. Zhang, W. Ouyang, X. Ren, H. Liang, H. Zheng, Y. Huang, J. Li, L. Bolund, K. Kristiansen, Y. Li, Y. Zhang, X. Zhang, R. Li, S. Li, H. Yang, R. Nielsen, J. Wang, J. Wang, Sequencing of 50 human exomes reveals adaptation to high altitude. *Science* **329**, 75–78 (2010). doi:10.1126/science.1190371 Medline

42. B. Vernot, A. B. Stergachis, M. T. Maurano, J. Vierstra, S. Neph, R. E. Thurman, J. A. Stamatoyannopoulos, J. M. Akey, Personal and population genomics of human regulatory variation. *Genome Res.* **22**, 1689–1697 (2012). doi:10.1101/gr.134890.111 Medline

43. D. G. Torgerson, A. R. Boyko, R. D. Hernandez, A. Indap, X. Hu, T. J. White, J. J. Sninsky, M. Cargill, M. D. Adams, C. D. Bustamante, A. G. Clark, Evolutionary processes acting on candidate cis-regulatory regions in humans inferred from patterns of polymorphism and divergence. *PLOS Genet.* **5**, e1000592 (2009). doi:10.1371/journal.pgen.1000592 Medline

44. F. Peng, G. Zhu, P. G. Hysi, R. J. Eller, Y. Chen, Y. Li, M. A. Hamer, C. Zeng, R. L. Hopkins, C. L. Jacobus, P. L. Wallace, A. G. Uitterlinden, M. A. Ikram, T. Nijsten, D. L. Duffy, S. E. Medland, T. D. Spector, S. Walsh, N. G. Martin, F. Liu, M. Kayser; International Visible Trait Genetics Consortium, Genome-Wide Association Studies Identify Multiple Genetic Loci Influencing Eyebrow Color Variation in Europeans. *J. Invest. Dermatol.* **139**, 1601–1605 (2019). doi:10.1016/j.jid.2018.12.029 Medline

45. R. L. Lamason, M.-A. P. K. Mohideen, J. R. Mest, A. C. Wong, H. L. Norton, M. C. Aros, M. J. Jurynec, X. Mao, V. R. Humphreville, J. E. Humbert, S. Sinha, J. L. Moore, P. Jagadeeswaran, W. Zhao, G. Ning, I. Makalowska, P. M. McKeigue, D. O'donnell, R. Kittles, E. J. Parra, N. J. Mangini, D. J. Grunwald, M. D. Shriver, V. A. Canfield, K. C. Cheng, SLC24A5, a putative cation exchanger, affects pigmentation in zebrafish and humans. *Science* **310**, 1782–1786 (2005). doi:10.1126/science.1116238 Medline

46. R. A. Sturm, D. L. Duffy, Human pigmentation genes under environmental selection. *Genome Biol.* **13**, 248 (2012). doi:10.1186/gb-2012-13-9-248 Medline

47. P. C. Sabeti, P. Varilly, B. Fry, J. Lohmueller, E. Hostetter, C. Cotsapas, X. Xie, E. H. Byrne, S. A. McCarroll, R. Gaudet, S. F. Schaffner, E. S. Lander, K. A. Frazer, D. G. Ballinger, D. R. Cox, D. A. Hinds, L. L. Stuve, R. A. Gibbs, J. W. Belmont, A. Boudreau, P. Hardenbol, S. M. Leal, S. Pasternak, D. A. Wheeler, T. D. Willis, F. Yu, H. Yang, C. Zeng, Y. Gao, H. Hu, W. Hu, C. Li, W. Lin, S. Liu, H. Pan, X. Tang, J. Wang, W. Wang, J. Yu, B. Zhang, Q. Zhang, H. Zhao, H. Zhao, J. Zhou, S. B. Gabriel, R. Barry, B. Blumenstiel, A. Camargo, M. Defelice, M. Faggart, M. Goyette, S. Gupta, J. Moore, H. Nguyen, R. C. Onofrio, M. Parkin, J. Roy, E. Stahl, E. Winchester, L. Ziaugra, D. Altshuler, Y. Shen, Z. Yao, W. Huang, X. Chu, Y. He, L. Jin, Y. Liu, Y. Shen, W. Sun, H. Wang, Y. Wang, Y. Wang, X. Xiong, L. Xu, M. M. Waye, S. K. Tsui, H. Xue, J. T. Wong, L. M. Galver, J. B. Fan, K. Gunderson, S. S. Murray, A. R. Oliphant, M. S. Chee, A. Montpetit, F. Chagnon, V. Ferretti, M. Leboeuf, J. F. Olivier, M. S. Phillips, S.

Roumy, C. Sallée, A. Verner, T. J. Hudson, P. Y. Kwok, D. Cai, D. C. Koboldt, R. D. Miller, L. Pawlikowska, P. Taillon-Miller, M. Xiao, L. C. Tsui, W. Mak, Y. Q. Song, P. K. Tam, Y. Nakamura, T. Kawaguchi, T. Kitamoto, T. Morizono, A. Nagashima, Y. Ohnishi, A. Sekine, T. Tanaka, T. Tsunoda, P. Deloukas, C. P. Bird, M. Delgado, E. T. Dermitzakis, R. Gwilliam, S. Hunt, J. Morrison, D. Powell, B. E. Stranger, P. Whittaker, D. R. Bentley, M. J. Daly, P. I. de Bakker, J. Barrett, Y. R. Chretien, J. Maller, S. McCarroll, N. Patterson, I. Pe'er, A. Price, S. Purcell, D. J. Richter, P. Sabeti, R. Saxena, S. F. Schaffner, P. C. Sham, P. Varilly, D. Altshuler, L. D. Stein, L. Krishnan, A. V. Smith, M. K. Tello-Ruiz, G. A. Thorisson, A. Chakravarti, P. E. Chen, D. J. Cutler, C. S. Kashuk, S. Lin, G. R. Abecasis, W. Guan, Y. Li, H. M. Munro, Z. S. Qin, D. J. Thomas, G. McVean, A. Auton, L. Bottolo, N. Cardin, S. Eyheramendy, C. Freeman, J. Marchini, S. Myers, C. Spencer, M. Stephens, P. Donnelly, L. R. Cardon, G. Clarke, D. M. Evans, A. P. Morris, B. S. Weir, T. Tsunoda, T. A. Johnson, J. C. Mullikin, S. T. Sherry, M. Feolo, A. Skol, H. Zhang, C. Zeng, H. Zhao, I. Matsuda, Y. Fukushima, D. R. Macer, E. Suda, C. N. Rotimi, C. A. Adebamowo, I. Ajayi, T. Aniagwu, P. A. Marshall, C. Nkwodimmah, C. D. Royal, M. F. Leppert, M. Dixon, A. Peiffer, R. Qiu, A. Kent, K. Kato, N. Niikawa, I. F. Adewole, B. M. Knoppers, M. W. Foster, E. W. Clayton, J. Watkin, R. A. Gibbs, J. W. Belmont, D. Muzny, L. Nazareth, E. Sodergren, G. M. Weinstock, D. A. Wheeler, I. Yakub, S. B. Gabriel, R. C. Onofrio, D. J. Richter, L. Ziaugra, B. W. Birren, M. J. Daly, D. Altshuler, R. K. Wilson, L. L. Fulton, J. Rogers, J. Burton, N. P. Carter, C. M. Clee, M. Griffiths, M. C. Jones, K. McLay, R. W. Plumb, M. T. Ross, S. K. Sims, D. L. Willey, Z. Chen, H. Han, L. Kang, M. Godbout, J. C. Wallenburg, P. L'Archevêque, G. Bellemare, K. Saeki, H. Wang, D. An, H. Fu, Q. Li, Z. Wang, R. Wang, A. L. Holden, L. D. Brooks, J. E. McEwen, M. S. Guyer, V. O. Wang, J. L. Peterson, M. Shi, J. Spiegel, L. M. Sung, L. F. Zacharia, F. S. Collins, K. Kennedy, R. Jamieson, J. Stewart; International HapMap Consortium, Genome-wide detection and characterization of positive selection in human populations. *Nature* **449**, 913–918 (2007). doi:10.1038/nature06250 Medline

48. G. Raposo, D. Tenza, D. M. Murphy, J. F. Berson, M. S. Marks, Distinct protein sorting and localization to premelanosomes, melanosomes, and lysosomes in pigmented melanocytic cells. *J. Cell Biol.* **152**, 809–824 (2001). doi:10.1083/jcb.152.4.809 Medline

49. K. Liu, Y. Jian, X. Sun, C. Yang, Z. Gao, Z. Zhang, X. Liu, Y. Li, J. Xu, Y. Jing, S. Mitani, S. He, C. Yang, Negative regulation of phosphatidylinositol 3-phosphate levels in early-to-late endosome conversion. *J. Cell Biol.* **212**, 181–198 (2016). doi:10.1083/jcb.201506081 Medline

50. S. K. Mallipattu, S. J. Horne, V. D'Agati, G. Narla, R. Liu, M. A. Frohman, K. Dickman, E. Y. Chen, A. Ma'ayan, A. B. Bialkowska, A. M. Ghaleb, M. O. Nandan, M. K. Jain, I. Daehn, P. Y. Chuang, V. W. Yang, J. C. He, Krüppel-like factor 6 regulates mitochondrial function in the kidney. *J. Clin. Invest.* **125**, 1347–1361 (2015). doi:10.1172/JCI77084 Medline

51. V. Delmas, S. Martinozzi, Y. Bourgeois, M. Holzenberger, L. Larue, Cre-mediated recombination in the skin melanocyte lineage. *Genesis* **36**, 73–80 (2003). doi:10.1002/gene.10197 Medline

52. B. Nabet, F. M. Ferguson, B. K. A. Seong, M. Kuljanin, A. L. Leggett, M. L. Mohardt, A. Robichaud, A. S. Conway, D. L. Buckley, J. D. Mancias, J. E. Bradner, K. Stegmaier, N. S. Gray, Rapid and direct control of target protein levels with VHL-recruiting dTAG molecules. *Nat. Commun.* **11**, 4687 (2020). doi:10.1038/s41467-020-18377-w Medline

53. S. R. Setty, D. Tenza, E. V. Sviderskaya, D. C. Bennett, G. Raposo, M. S. Marks, Cell-specific ATP7A transport sustains copper-dependent tyrosinase activity in melanosomes. *Nature* **454**, 1142–1146 (2008). doi:10.1038/nature07163 Medline

54. B. J. Bowman, M. E. McCall, R. Baertsch, E. J. Bowman, A model for the proteolipid ring and bafilomycin/concanamycin-binding site in the vacuolar ATPase of Neurospora crassa. *J. Biol. Chem.* **281**, 31885–31893 (2006). doi:10.1016/S0021-9258(19)84103-0 Medline

55. P. W. Halcrow, J. D. Geiger, X. Chen, Overcoming Chemoresistance: Altering pH of Cellular Compartments by Chloroquine and Hydroxychloroquine. *Front. Cell Dev. Biol.* **9**, 627639 (2021). doi:10.3389/fcell.2021.627639 Medline

56. S. J. Huh, Y.-L. Chen, S. L. Friedman, J. Liao, H.-J. S. Huang, W. K. Cavenee, G. P. Robertson, KLF6 Gene and early melanoma development in a collagen I-rich extracellular environment. *J. Natl. Cancer Inst.* **102**, 1131–1147 (2010). doi:10.1093/jnci/djq218 Medline

57. D. Cai, J. Zhao, Q. Sun, Krüppel-like factor 6 in the progression and prognosis of malignant melanoma. *J. Int. Med. Res.* **42**, 184–190 (2014). doi:10.1177/0300060513499092 Medline

58. G. Ito, M. Uchiyama, M. Kondo, S. Mori, N. Usami, O. Maeda, T. Kawabe, Y. Hasegawa, K. Shimokata, Y. Sekido, Krüppel-like factor 6 is frequently down-regulated and induces apoptosis in non-small cell lung cancer cells. *Cancer Res.* **64**, 3838–3843 (2004). doi:10.1158/0008-5472.CAN-04-0185 Medline

59. G. Narla, K. E. Heath, H. L. Reeves, D. Li, L. E. Giono, A. C. Kimmelman, M. J. Glucksman, J. Narla, F. J. Eng, A. M. Chan, A. C. Ferrari, J. A. Martignetti, S. L. Friedman, KLF6, a candidate tumor suppressor gene mutated in prostate cancer. *Science* **294**, 2563–2566 (2001). doi:10.1126/science.1066326 Medline

60. G. Narla, S. Kremer-Tal, N. Matsumoto, X. Zhao, S. Yao, K. Kelley, M. Tarocchi, S. L. Friedman, In vivo regulation of p21 by the Krüppel-like factor 6 tumor-suppressor gene in mouse liver and human hepatocellular carcinoma. *Oncogene* **26**, 4428–4434 (2007). doi:10.1038/sj.onc.1210223 Medline

61. B. Zhang, D. D. Guo, J. Y. Zheng, Y. A. Wu, Expression of KLF6-SV2 in colorectal cancer and its impact on proliferation and apoptosis. *Eur. J. Cancer Prev.* **27**, 20–26 (2018). doi:10.1097/CEJ.0000000000000410 Medline

62. K. Double, W. Maruyama, M. Naoi, M. Gerlach, P. Riederer, in *Melanins and Melanosomes*, J. Borovansky, P. A. Riley, Eds. (Wiley-Blackwell, 2011), pp. 225–246.

63. Y. Yamaguchi, V. J. Hearing, Melanocytes and their diseases. *Cold Spring Harb. Perspect. Med.* **4**, a017046 (2014). doi:10.1101/cshperspect.a017046 Medline

64. V. K. Bajpai *et al.*, A genome-wide genetic screen uncovers novel determinants of human pigmentation, Zenodo (2023); https://doi.org/10.5281/zenodo.7991815.

65. V. K. Bajpai, P. Mistriotis, Y. H. Loh, G. Q. Daley, S. T. Andreadis, Functional vascular smooth muscle cells derived from human induced pluripotent stem cells via mesenchymal stem cell intermediates. *Cardiovasc. Res.* **96**, 391–400 (2012). doi:10.1093/cvr/cvs253 Medline

66. V. K. Bajpai, L. Kerosuo, G. Tseropoulos, K. A. Cummings, X. Wang, P. Lei, B. Liu, S. Liu, G. K. Popescu, M. E. Bronner, S. T. Andreadis, Reprogramming Postnatal Human Epidermal Keratinocytes Toward Functional Neural Crest Fates. *Stem Cells* **35**, 1402–1415 (2017). doi:10.1002/stem.2583 Medline

67. S. Vaidyanathan, A. A. Salahudeen, Z. M. Sellers, D. T. Bravo, S. S. Choi, A. Batish, W. Le, R. Baik, S. de la O, M. P. Kaushik, N. Galper, C. M. Lee, C. A. Teran, J. H. Yoo, G. Bao, E. H. Chang, Z. M. Patel, P. H. Hwang, J. J. Wine, C. E. Milla, T. J. Desai, J. V. Nayak, C. J. Kuo, M. H. Porteus, High-Efficiency, Selection-free Gene Repair in Airway Stem Cells from Cystic Fibrosis Patients Rescues CFTR Function in Differentiated Epithelia. *Cell Stem Cell* **26**, 161–171.e4 (2020). doi:10.1016/j.stem.2019.11.002 Medline

68. J. Hosoi, E. Abe, T. Suda, T. Kuroki, Regulation of melanin synthesis of B16 mouse melanoma cells by 1 alpha, 25-dihydroxyvitamin D3 and retinoic acid. *Cancer Res.* **45**, 1474–1478 (1985). Medline

69. N. Kollias, *The Spectroscopy of Human Melanin Pigmentation in Melanin: Its Role in Human Photoprotection* (Valdenmar Publishing Co., 1995).

70. S. Abounit, L. Bousset, F. Loria, S. Zhu, F. de Chaumont, L. Pieri, J.-C. Olivo-Marin, R. Melki, C. Zurzolo, Tunneling nanotubes spread fibrillar α-synuclein by intercellular trafficking of lysosomes. *EMBO J.* **35**, 2120–2138 (2016). doi:10.15252/embj.201593411 Medline

71. S. Bolte, F. P. Cordelières, A guided tour into subcellular colocalization analysis in light microscopy. *J. Microsc.* **224**, 213–232 (2006). doi:10.1111/j.1365-2818.2006.01706.x Medline

72. E. M. M. Manders, F. J. Verbeek, J. A. Aten, Measurement of co-localization of objects in dual-colour confocal images. *J. Microsc.* **169**, 375–382 (1993). doi:10.1111/j.1365-2818.1993.tb03313.x Medline

73. E. Eden, R. Navon, I. Steinfeld, D. Lipson, Z. Yakhini, GOrilla: A tool for discovery and visualization of enriched GO terms in ranked gene lists. *BMC Bioinformatics* **10**, 48 (2009). doi:10.1186/1471-2105-10-48 Medline

74. F. Supek, M. Bošnjak, N. Škunca, T. Šmuc, REVIGO summarizes and visualizes long lists of gene ontology terms. *PLOS ONE* **6**, e21800 (2011). doi:10.1371/journal.pone.0021800 Medline

75. N. Sinnott-Armstrong, S. Naqvi, M. Rivas, J. K. Pritchard, GWAS of three molecular traits highlights core genes and pathways alongside a highly polygenic background. *eLife* **10**, e58615 (2021). doi:10.7554/eLife.58615 Medline

76. 1000 Genomes Project Consortium, A global reference for human genetic variation. *Nature* **526**, 68–74 (2015). doi:10.1038/nature15393 Medline

77. P. Danecek, A. Auton, G. Abecasis, C. A. Albers, E. Banks, M. A. DePristo, R. E. Handsaker, G. Lunter, G. T. Marth, S. T. Sherry, G. McVean, R. Durbin; 1000 Genomes Project Analysis Group, The variant call format and VCFtools. *Bioinformatics* **27**, 2156–2158 (2011). doi:10.1093/bioinformatics/btr330 Medline

78. A. Frankish, M. Diekhans, A.-M. Ferreira, R. Johnson, I. Jungreis, J. Loveland, J. M. Mudge, C. Sisu, J. Wright, J. Armstrong, I. Barnes, A. Berry, A. Bignell, S. Carbonell Sala, J. Chrast, F. Cunningham, T. Di Domenico, S. Donaldson, I. T. Fiddes, C. García Girón, J. M. Gonzalez, T. Grego, M. Hardy, T. Hourlier, T. Hunt, O. G. Izuogu, J. Lagarde, F. J. Martin, L. Martínez, S. Mohanan, P. Muir, F. C. P. Navarro, A. Parker, B. Pei, F. Pozo, M. Ruffier, B. M. Schmitt, E. Stapleton, M.-M. Suner, I. Sycheva, B. Uszczynska-Ratajczak, J. Xu, A. Yates, D. Zerbino, Y. Zhang, B. Aken, J. S. Choudhary, M. Gerstein, R. Guigó, T. J. P. Hubbard, M. Kellis, B. Paten, A. Reymond, M. L. Tress, P. Flicek, GENCODE reference annotation for the human and mouse genomes. *Nucleic Acids Res.* **47**, D766–D773 (2019). doi:10.1093/nar/gky955 Medline

79. G. Bhatia, N. Patterson, S. Sankararaman, A. L. Price, Estimating and interpreting FST: The impact of rare variants. *Genome Res.* **23**, 1514–1521 (2013). doi:10.1101/gr.154831.113 Medline
